# Overview of porous magnesium-based scaffolds: development, properties and biomedical applications

**DOI:** 10.1088/2752-5724/ad9493

**Published:** 2025-01-02

**Authors:** Amir Motaharinia, Jaroslaw W Drelich, Safian Sharif, Ahmad Fauzi Ismail, Farid Naeimi, Alexandra Glover, Mahshid Ebrahiminejad, Hamid Reza Bakhsheshi-Rad

**Affiliations:** 1 Advanced Materials Research Center, Department of Materials Engineering, Najafabad Branch, Islamic Azad University, Najafabad, Iran; 2 Department of Materials Science and Engineering, Michigan Technological University, Houghton, MI 49931, United States of America; 3 Advanced Manufacturing Research Group, Faculty of Mechanical Engineering, Universiti Teknologi Malaysia, Johor Bahru 81310, Johor, Malaysia; 4 Advanced Membrane Technology Research Center (AMTEC), Universiti Teknologi Malaysia, Johor Bahru 81310, Johor, Malaysia; 5 Department of Mechanical Engineering, Najafabad Branch, Islamic Azad University, Najafabad, Iran

**Keywords:** magnesium-based alloys, porous scaffolds, bone tissue engineering, GASAR technique, space holder, additive manufacturing, biocompatibility

## Abstract

Magnesium (Mg) and its alloys are revolutionizing the field of interventional surgeries in the medical industry. Their high biocompatibility, biodegradability, and a similar elastic modulus to natural bone make porous Mg-based structures potential candidates for orthopedic implants and tissue engineering scaffolding. However, fabricating and machining porous Mg-based structures is challenging due to their complexity and difficulties in achieving uniform or gradient porosity. This review aims to thoroughly explore various fabrication procedures used to create metallic scaffolds, with a specific focus on those made from Mg-based alloys. Both traditional manufacturing techniques, including the directional solidification of metal-gas eutectic technique, pattern casting, methods using space holders, and modern fabrication methods, which are based on additive manufacturing, are covered in this review article. Furthermore, the paper highlights the most important findings of recent studies on Mg-based scaffolds in terms of their microstructure specifications, mechanical properties, degradation and corrosion behavior, antibacterial activity, and biocompatibility (both *in vivo* and *in vitro*). While extensive research has been conducted to optimize manufacturing parameters and qualities of Mg-based scaffolds for use in biomedical applications, specifically for bone tissue engineering applications, further investigation is needed to fabricate these scaffolds with specific properties, such as high resistance to corrosion, good antibacterial properties, osteoconductivity, osteoinductivity, and the ability to elicit a favorable response from osteoblast-like cell lines. The review concludes with recommendations for future research in the field of medical applications.

**Abbreviations**
(NH_2_)_2_CO: Carbamide (urea)
ALP: Alkaline phosphatase
BG: Bioactive glass
BJP: Binder jet printing
C_10_H_16_: Camphene
CE: Corrosion environment
CFS: Compressive flexure strength
CR: Corrosion rate
CSC: Cold spray coating
CYS: Compressive yield strength
Cu: Copper
DED: Direct energy deposition
DMEM: Dulbecco’s modified Eagle’s medium
E-PBF: Electron beam powder bed fusion
EA: Energy absorption
FBS: Fetal bovine serum
Fe: Iron
GJIC: Gap junction intercellular communication
H&E: Hematoxylin and eosin
HIV: Human immunodeficiency virus
IC: Investment casting
K_2_CO_3_: Potassium carbonate
MCMZ: Mg–Ca–Mn–Zn
MPS: Median pore size
MZ: Mg–Zn
MgO: Magnesium oxide
Mo: Molybdenum
NaCl: Sodium chloride
PBF: Powder bed fusion
PCL: Polycaprolactone
PPF: Polypropylene fibers
PS: Pore size
RE
: Rare earth element
SBF: Simulated body fluid
SLM: Selective laser melting
TA: Tannic acid
TCP: Tricalcium phosphate
TWSH: Titanium wire space holder
UCS: Ultimate compressive strength
VIC: Vacuum-assisted investment casting
VRF: Viscose rayon fiber
YS: Yield strength
Zn: Zinc
rBMSCs: Rat bone marrow-derived mesenchymal stem cells
3D: Three-dimensional
AM: Additive manufacturing
BJ: Binder jetting
C_12_H_22_O_11_: Saccharose
CF
: Corrosion fatigue
CPS: Compressive peak stress
CS: Compressive strength
CT: Computed tomography
CaP: Calcium phosphate
DC: Doxycycline
*E. coli*
: *Escherichia coli*
EAC: Energy absorption capacity
EBM: Electron beam melting
FDM: Fused deposition modeling
GASAR: Directional solidification of metal-gas eutectic
Gel: Gelatin
HA: Hydroxyapatite
HF: Hydrofluoric acid
L-PBF: Laser beam powder bed fusion
MCT: Mg–Ca–TiO_2_
MSCs: Mesenchymal stem cells
Mg: Magnesium
MgSO_4_: Magnesium sulfate
NH_4_HCO_3_: Ammonium bicarbonate
OPN: Osteopontin
PBS: Phosphate buffered saline
PEO: Plasma electrolytic oxidation
PLA: Polylactic acid
PMMA: Polymethylmethacrylate
PPI: Pore per inch
PU: Polyurethane
*S. aureus*
: *Staphylococcus aureus*
SEM: Scanning electron microscope
SPS: Spark plasma sintering
TC: Tetracycline
Ti: Titanium
UTS: Ultimate tensile strength
VIM: Vacuum induction melting machine
WAAM: Wire arc additive manufacturing
WOS: Web of science
Zeo: Zeolite
*μ*CT: Micro-computed tomography

## 1. Introduction

**Figure 1.**
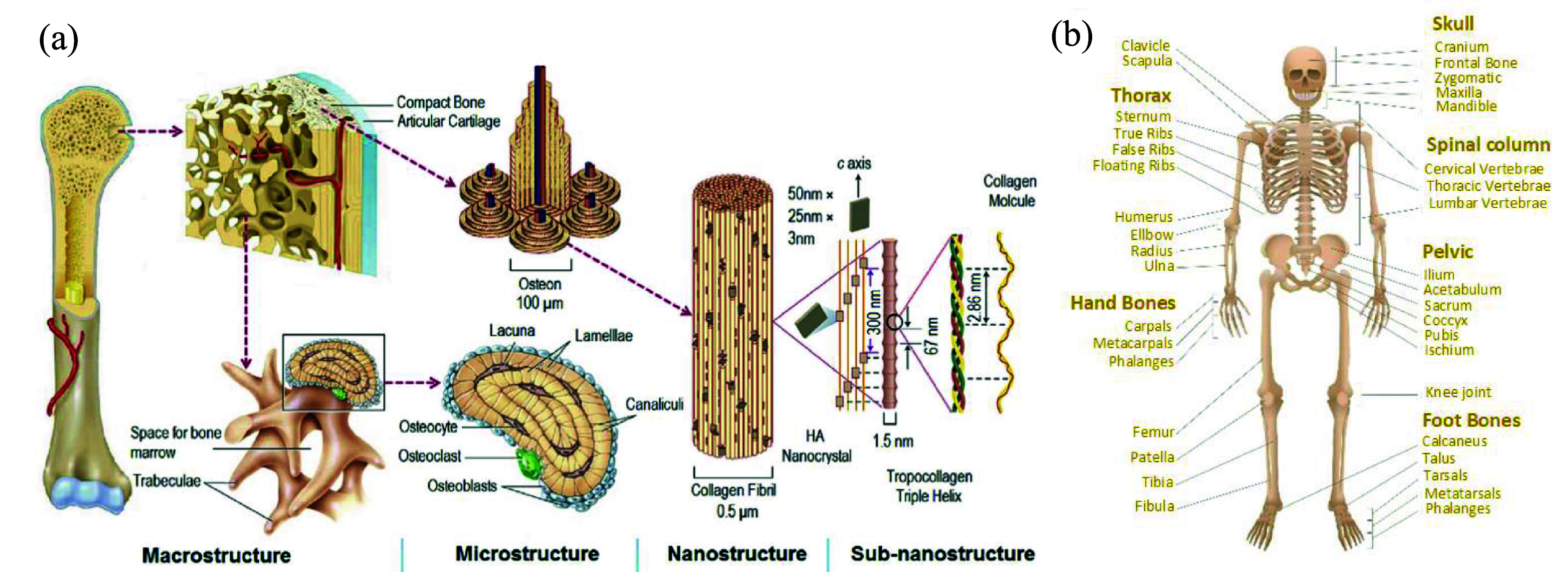
(a) Arranged hierarchically structure of bone. Reprinted from [1], Copyright © 2016 Elsevier Ltd All rights reserved. (b) Bones in human body (courtesy of iStock). Reproduced with permission from iStock.com/[PeterHermesFurian].

Bone is a vital and multifunctional organ with a hierarchical structural arrangement as shown in figure 1(a) [1]. The 206 bones in the adult human body have multiple vital functions (figure 1(b)), which include supporting and aiding movement while bearing weight, producing blood cells (hematopoiesis), protecting key organs including the brain and heart, and storing minerals and growth hormones [2, 3]. Bone undergoes continuous remodeling under normal physiological settings and possesses a robust regenerating ability to respond to fractures.

In cases involving the removal of bone tumors, major abnormalities, or infections, the natural process of bone repair can be disrupted [5]. This may prevent the affected area from fully regenerating on its own. Surgery becomes necessary when the extent of the defect surpasses the body’s ability to heal itself. Large and critical bone abnormalities pose a clinical challenge that affects millions of people worldwide [3]. A bone autograft is considered the preferred option for bone defect regeneration due to its ability to provide scaffolding that promotes bone growth while also containing cells that stimulate bone formation and natural growth factors. Bone autograft also has a high rate of integration with early blood vessel formation [6–8]. However, the use of bone autograft is significantly limited by increased fracture rates, adverse effects on the donor site, and limited availability [5, 6]. Therefore, it is essential to develop biodegradable porous scaffolds with suitable mechanical properties, biocompatibility, osteointegration, osteoconductivity, and osteoinductivity for musculoskeletal tissue engineering to meet the increasing demand for orthopedic implants [9, 10].

Biodegradable materials are essential because the scaffolds serve as temporary frameworks that guide cell growth and tissue formation. These biodegradable materials should be gradually broken down over time at a similar rate as the bone tissue healing time, allowing the body’s cells to replace it [11, 12]. Permanent implants not only impede this natural process but also have the potential to cause a range of long-term consequences, including chronic inflammation, blood clot formation, and vascular anomalies if not removed by a second surgery operation [13]. In addition, using biodegradable scaffolds can minimize the risk of inflammation, rejection, or foreign body responses, especially in long-term uses [12, 14]. Bone implants made of bio-inert materials like stainless steel do not properly integrate with the bone. Instead, they become encapsulated by fibrous tissue shortly after implantation. Also, because their elastic properties do not match those of natural bone, they cause stress-shielding, leading to implant failure. Alternatively, biopolymeric and bioceramic scaffolds can be used for bone tissue engineering, but only for small defects and in non-load-bearing sites due to the low mechanical strength of polymers and the limited toughness and brittleness of ceramics [1, 15, 16]. To ensure better biocompatibility and biodegradability, it is preferable to use metallic scaffolds, particularly in load-bearing applications.

### 1.1. Magnesium as a biodegradable medical implant

Biodegradable metals are generally classified into three main categories: iron (Fe)-based, zinc (Zn)-based, and magnesium (Mg)-based [17, 18]. The densities and characteristics of each category are outlined in table 1. Recently, new research [19] evaluated the degradation behavior and biocompatibility of Mo as a new biodegradable metallic candidate for biomedical applications.

**Table 1.** Density values and remarks of cancellous bone, cortical bone, and available biodegradable metals.

Metal	Density (g cm^−3^)	Elastic modulus (GPa)	Compressive strength (MPa)	Remarks	References
Cortical bone	1.8–2.1	3–20	130–180	• The porosity of trabecular bone varies between 40% and 95%, while that of cortical bone is between 5% and 15%. • Cortical bone can be found in the diaphysis of long bones as well as in the metaphyses and epiphyses, where it surrounds the trabecular bone in the shape of a thin shell. The vertebrae also include trabecular bone. • Cortical bone has stronger and more compressive/tensile moduli in its longitudinal direction compared to its radial and circumferential orientations. • The compressive strength and Young modulus of cortical bone are measured between 130 and 180 GPa and 3–20 GPa, respectively, but the values for trabecular bones are between 0.8 and 11.6 MPa and 0.01–2 GPa, respectively.	[20, 21]
Trabecular bone	—	0.01–2	0.8–11.6		

Fe	7.87	200–205	—	• Provides sufficient time and mechanical support for new bone ingrowth in initial stages. • Even though Fe is hardly ever found in natural bone, Fe is crucial for the development of new bone. • Too slow degradation rate (from 2–3 to several years, depending on size). • Direct cell seeding on pure Fe replacements results in cytotoxicity. • High elastic modules compared to those of natural bone, which leads to a stress-shielding effect, specifically in long-term uses. • Ferromagnetic behavior.	[14, 22–25]

Zn	7.14	90–100	—	• Adequate corrosion rate to the rate at which bone tissue regenerates. • A vital metallic element for the human body that can promote bone formation. • Good antibacterial activity. • The primary obstacle that restricts the widespread use of Zn and its alloys in biomedical applications is having lower mechanical strength compared to other materials like Mg or Fe, which can lead to issues with maintaining the structural integrity of the Zn-based scaffold over time.	[13, 26–28]

Mg	1.74	35–45	100–250	• Closer Young modulus (41–45 GPa) to the natural human bone (3–20 GPa) compared to Fe (211.4 GPa) and Zn (90 GPa), which reduces the risk of stress-shielding, especially in long-term uses. • A crucial metallic element in the human body, it participates in a variety of enzymatic and metabolic processes. • Easy machinability • Good capability in enhancing osteoblast activity. • Providing adequate osteoconductivity. • Degradation by-products, are typically eliminated from the bloodstream and expelled through urine by the kidneys. • Fast degradation rate in biological environment (1–4 months). • Harmful hydrogen gas evolution might inhibit the integration of bone and scaffold.	[29–34]

**Figure 2.**
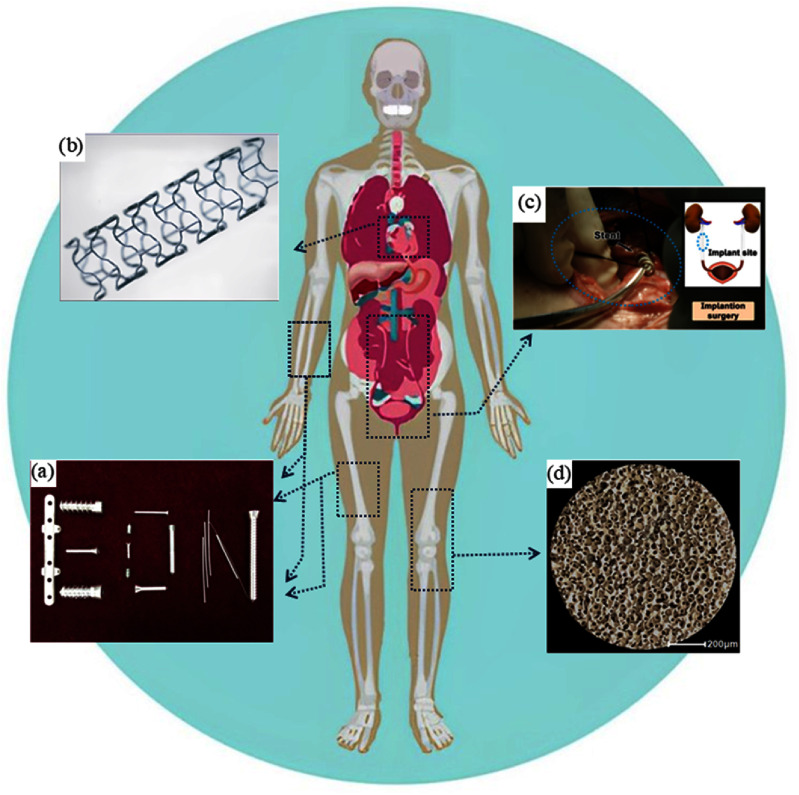
The exemplary Mg-based biomedical applications include: (a) the optical imagery of Mg-based implants for potential orthopedic applications. Reprinted from [58], © 2016 Published by Elsevier Ltd. (b) Cardiovascular Mg stent. Reprinted from [59], Copyright © 2007 Elsevier Ltd All rights reserved. (c) ZJ41 Mg-based urethral stent implantation. Reprinted from [40], © 2020 Published by Elsevier Ltd on behalf of Acta Materialia Inc. (d) Optical micrograph of as-prepared porous Mg-based (Mg–Zn–Ca) scaffold for bone tissue engineering application. Reproduced from [60]. CC BY 4.0.

A study by Xu *et al* [35], using data from Mg-based alloys research literature sourced from the WOS database between 2008 and 2018, showed that the publication growth rate for Mg-based alloys exceeded the overall growth rate of alloy research papers during this time. In another evaluation in 2021 [36], over 4000 studies related to Mg and Mg alloys were published and indexed in the WoS Core Collection database this year, focusing on the microstructure, mechanical characteristics, and corrosion of Mg-based alloys. Furthermore, an assessment by Abbott in 2015 [37] indicates that global Mg production has significantly increased since 2000, with China as the predominant producer. Since a resurgence of interest in the late 1990s, Mg and its alloys have received increased study focus in the field of biomaterials. Based on the information provided in table 1, Mg and Mg-based alloys seem to be the most promising option for fabricating biodegradable metallic implants. Mg-based porous structures (scaffolds) have been used as bone and cartilage [38] tissue engineering scaffolds and in stent applications such as cardiovascular [39], urethral [40], and esophageal [41] stents. Additionally, Mg-based biomaterials with high-density structures are considered as potential candidates for temporary craniofacial and orthopedic applications such as plates, screws, nails, and pins [42–45]. Despite numerous animal studies [42, 44, 46, 47] examining the safety and efficacy of Mg-based temporary craniofacial and orthopedic implants, the lack of clinical investigations remains the primary limitation to their widespread clinical application. MAGNEZIX^®^ CS (composed of MgYReZr) [48–50] and Mg–Ca–Zn [51, 52] screws are recognized as Mg-based orthopedic implants that have undergone extensive clinical evaluation. In the context of using cardiovascular stents, it is challenging to achieve sufficient visibility during angioplasty, especially in complex cases and scenarios where stent visualization is compromised. This is particularly true for stents with very thin struts, in obese patients, or in highly calcified vessels, which have low-density mesh-like structures [53–55]. Mg-based stents exhibit comparatively low radiopacity relative to other prevalent biodegradable and permanent metallic stents, mainly due to their lower atomic number and density. Utilizing radiopaque markers like tantalum can increase the radiopacity of Mg-based stents [56, 57]. Figure 2 provides illustrations of various biomedical applications of Mg and Mg-based alloys [40, 58–60].

The objective of this investigation is to deliver a thorough analysis of Mg-based scaffolds for applications in bone tissue engineering. Here, the following text provides a detailed discussion of some of the features of Mg and Mg-based alloys in this regard, including both advantages (such as adequate mechanical properties and enhanced osteoblast activity) and disadvantages (such as a rapid degradation rate in the biological environment), to provide a more comprehensive understanding.

### 1.2. Characteristics of Mg as a medical implant

#### 1.2.1. Biocompatibility and cell behavior.

Mg demonstrates excellent biocompatibility [62]. Not only do Mg ions released from Mg-based scaffolds not cause toxicity in the human body, but they also have the ability to promote the recovery of bone tissue [62, 63]. Furthermore, Mg ions play a significant role in the differentiation and expression of osteogenic markers, as well as influencing the behavior and activity of osteoblasts (which are the cells responsible for bone formation) [63]. Mg is a vital component for promoting osteoblastic activity, which is crucial for the development and remodeling of bones where it acts as a stimulant for osteoblasts, promoting their proliferation [64–68]. Research has shown that the presence of Mg^2+^ ions has a significant impact on the survival of human osteoblasts, as well as their ALP activity and osteocalcin levels [64, 67, 68]. Mg^2+^ ions have the ability to stimulate MSCs, transforming them into bone cells, which improves bone production and growth [69]. Additionally, Mg significantly enhances GJIC among osteoblasts, which is crucial for their coordinated function. A deficiency in Mg can directly impact osteoporosis by influencing the development of crystals and bone cells [64, 68–70]. Bioactive materials coated on the surface of Mg scaffolds can further promote osteoblastic activity and the ingrowth of new tissue [71, 72]. The biocompatibility and cellular behavior of Mg-based scaffolds are impacted not only by the potential of Mg ions but also by additional scaffold parameters, including corrosion behavior, structural specifications, and antibacterial characteristics. The impact of these elements is outlined in the subsequent sections.

#### 1.2.2. Degradation and corrosion mechanism.

As mentioned in table 1, the fast CR of Mg and Mg-based alloys in physiological settings presents obstacles to their effective utilization as implants, particularly in orthopedic applications, due to a rapid loss in mechanical stability [73–76]. The following sub-section delves into the complex relationship between Mg and biological systems, elucidating its behavior and underlying mechanisms.

When a Mg based biomaterial is implanted in the body, it slowly dissolves over time. This process, known as the anodic dissolution reaction (equation (1)), results in the discharge of Mg ions into the body and the surrounding tissue. This process is important for biodegradable implants because it allows for gradual replacement by new tissue. However, in the case of Mg and Mg-based materials, rapid breakdown may cause cytotoxic effects and inflammation due to the increased local accumulation of alkaline ions during the initial stages of degradation [77, 78],

\begin{align*}{\text{Mg}} \leftrightarrow {\text{M}}{{\text{g}}^{{\text{2} + }}} + {\text{2}}{{\text{e}}^{\text{-}}}\left( {{\text{anodic reaction}}} \right).\end{align*}

The next stage is a cathodic reaction (equation (2)), resulting in the evolution of hydrogen (H_2_) gas. The high volume of hydrogen production in this stage is considered a critical challenge as it can prohibit the integration of the implant by damaging surrounding tissue and causing infection in the implant site. Balancing hydrogen evolution is critical for implant longevity [77, 78],

\begin{align*}{\text{2}}{{\text{H}}_{\text{2}}}{\text{O }+ \text{2}}{{\text{e}}^{\text{-}}} \leftrightarrow {{\text{H}}_{\text{2}}} + {\text{2O}}{{\text{H}}^{\text{-}}}\left( {{\text{cathodic reaction}}} \right).\end{align*}

When Mg ions react with water (H_2_O), they form magnesium hydroxide (Mg(OH)_2_) and release hydrogen gas (equations (3) and (4)). If there is too much Mg^2+^ present, it can reduce the crystallization of HA and impede the mineralization process during the early stages of implantation [77–79],

\begin{align*}{\text{Mg} + \text{2}}{{\text{H}}_{\text{2}}}{\text{O}} &amp; \leftrightarrow {\text{M}}{{\text{g}}^{{\text{2} + }}} + {{\text{H}}_{\text{2}}}{ + \text{2O}}{{\text{H}}^{\text{-}}}\left( {{\text{overall reaction}}} \right)\end{align*}

\begin{align*}{\text{Mg} + \text{2O}}{{\text{H}}^{\text{-}}} &amp; \leftrightarrow {\text{Mg}}{\left( {{\text{OH}}} \right)_{\text{2}}}\left( {{\text{product formation}}} \right).\end{align*}

**Figure 3.**
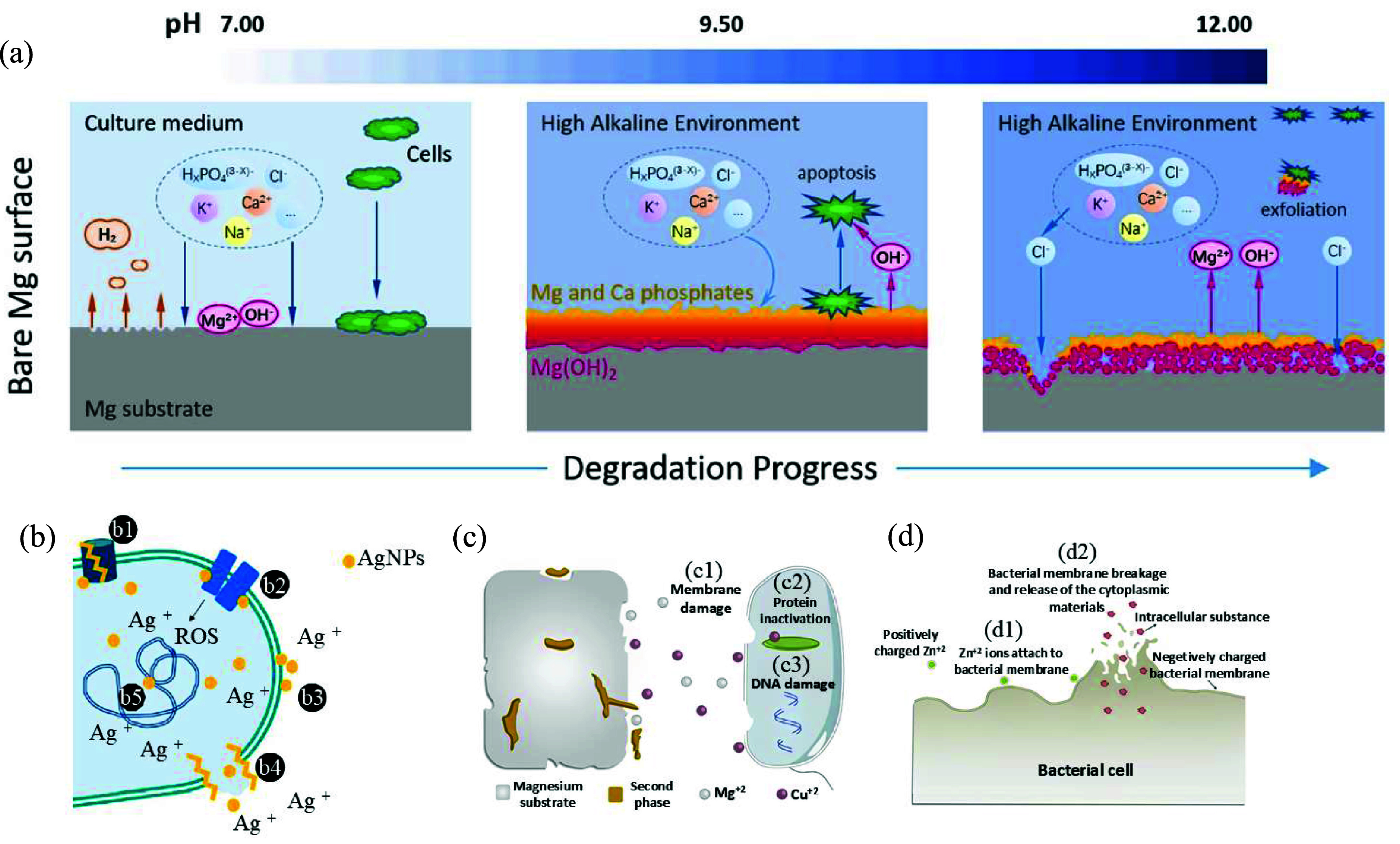
(a) Schematic representation depicting the procedure for deterioration and the biological characteristics of pure Mg. Reprinted from [81], © 2021 Elsevier B.V. All rights reserved. (b) An illustration of the antibacterial activity of Ag nanoparticles (AgNPs) including: (b1) alteration of efflux pumps, (b2) interruption of membrane proteins and electron transport chains, (b3) accumulation in the membrane influencing permeability, (b4) membrane rupture and intracellular content leakage, and (b5) interaction and damage in DNA. Reproduced from [83]. CC BY 4.0. (c) The antibacterial mechanism of Cu ions, and (d) the antibacterial mechanism of Zn ions. Reprinted from [84], © 2020 The Author(s). Published by Elsevier Ltd.

However, Mg(OH)_2_ creates a porous layer and can be transformed into soluble MgCl_2_. Over time, the hydroxide layer that provides protection breaks apart [80]. After the passive layer forms, a coating based on CaP is subsequently formed. This provides sites for the coating to initiate and develop by using calcium ions (Ca^2+^) and phosphate ions (PO_4_^3–^) from the nearby solution [81]. The reduction in the degradation rate of the scaffolds is due to the production of degradation products with lower solubility. With continued immersion, a balance is established between the creation and breakdown of corrosion substances (figure 3(a) [81]). The composition of bodily fluids is also highly significant. Chloride (Cl^–^) and sulfate (SO_4_^2–^) ions expedite corrosion [82]. Proteins and chemical compounds in biological fluids also influence the creation and composition of the surface film. Establishing a protective oxide layer (MgO) on the surface is vital for enhancing the corrosion resistance of Mg alloys by creating a surface film [74, 76]. The corrosion behavior of magnesium-based scaffolds is closely linked to their antibacterial properties. This significant relationship is further discussed in the following section.

#### 1.2.3. Antibacterial properties.

Implant infections can be caused by various opportunistic pathogens, such as bacteria [86]. The implant type and the location of insertion determine which pathogens are involved. These adaptable pathogens are effective because they can quickly attach to almost any biomaterial surface and survive in the challenging host environment. When bacteria colonize implant surfaces and form biofilms, they create an endangered environment that encourages long-term infection [86, 87]. Moreover, research has established that bacteria employ phenotypic and genetic tactics to innately defend against antibiotics and develop mechanisms to improve resistance to antibacterial agents [88, 89]. New research has found that Mg has strong antibacterial properties due to its high alkalinity when it breaks down. However, increasing the corrosion resistance of Mg and Mg-based alloys reduces their alkalinity and thus weakens their antibacterial effectiveness [84]. To address this, it is recommended to use antibacterial elements as alloying additions or coatings to enhance both the corrosion resistance and antibacterial properties of Mg-based alloys. Silver ions (Ag^2+^), zinc ions (Zn^2+^), and copper ions (Cu^2+^) are known for their antibacterial qualities and could be useful additions to Mg alloys [84, 90–93]. The antibacterial mechanisms of AgNPs, Zn^2+^, and Cu^2+^ ions are illustrated in figure 3(b) [83], figures 3(c) and (d) [84].

#### 1.2.4. Porosity and pore geometry.

Recent investigations have shown that the structural characteristics of porous scaffolds utilized in bone tissue engineering and bone substitution are vital in determining the cellular reaction and the speed of bone tissue renewal [94, 95]. Crucially, research has demonstrated that the rate at which tissue regenerates is higher on curved surfaces, particularly concave ones, compared to convex and flat surfaces [96, 97]. It is important to note that the ideal range of pore size in scaffolds for bone tissue engineering should be between 100 *μ*m and 600 *μ*m [11]. This range enables bone cells, like osteoblasts, to grow quickly and differentiate efficiently within the scaffolds. Scaffolds with larger pore diameters may compromise stability and mechanical properties, while those with smaller diameters (less than 100 *μ*m) restrict cell migration and nutrient flow. Additionally, the porosity of scaffolds should exceed 50% [11, 98–100]. Porous Mg-based structures with interconnected and isolated pore patterns can be used in biomedical applications. For load-bearing sites requiring high mechanical strength and EA characteristics, porous Mg-based structures with closely spaced pores can demonstrate more suitable properties [101]. However, interconnectivity is a crucial property in bone tissue engineering scaffolds, as it plays a vital role in regulating cell migration, blood vessel invasion, and the transportation of nutrients and waste [100, 102]. The fabrication process and their parameters dictate the structural properties of scaffolds.

### 1.3. Mg-based scaffolds fabrication techniques

When considering fabrication techniques, several methods have been developed for producing Mg-based scaffolds. These techniques include GASAR technique [103–105], pattern casting (IC) [106, 107], techniques based on utilizing space holders [108–110], and AM methods [27, 28]. However, maintaining precise control over the pore size, morphology, and interconnectivity (which are vital for ensuring the biomechanical integrity and anticipated biological reactions) is challenging in the case of traditional (non-AM) techniques [5, 16, 17].

The purpose of this article is to provide a summary of different fabrication techniques that have been used to create Mg-based scaffolds for bone tissue engineering applications. Additionally, it aims to offer a comprehensive review of important findings related to the properties and specifications of these Mg-based scaffolds. This includes details on microstructure, mechanical properties, corrosion behavior, cell response, antibacterial activity, and *in vivo* evaluations.

## 2. Fabrication techniques and the properties of Mg-based scaffolds

As mentioned before, various manufacturing methods have been used to fabricate porous Mg structures for biomedical applications, especially bone tissue engineering scaffolds. Each fabrication technology has its own advantages and limitations. This section provides a description of the fabrication procedures used and reviews the most important findings related to the specifications and characteristics of Mg-based scaffolds fabricated using these production techniques.

### 2.1. GASAR technique

The GASAR process involves creating regular porous metals with aligned pores by taking advantage of the difference in gas solubility between liquid and solid metals [111–113]. This is achieved by exposing molten metals saturated with gas, like hydrogen, to controlled solidification. Directional solidification can take different forms, such as the Bridgman process [114] where solidification starts at one end of the crucible and proceeds upward, or parallel solidification, which starts at the walls of the casting and progresses perpendicular to that surface. The GASAR process requires melting a substance in the presence of a gas (like hydrogen) to fully saturate it with the gas, and then solidifying it in a specific direction while closely controlling the thermodynamic and kinetic factors [113, 115, 116]. The necessary gas can be locally generated by subjecting fillers combined with the molten metal, such as VRFs and PPFs to pyrolysis [117, 118]. An inherent drawback of the GASAR process in producing metallic foams is the formation of unwanted substances inside the scaffold structure, which may change its properties and restrict its potential applications [118]. This approach enables accurate regulation of the porosity, pore size, and distribution of the oriented pore structure through the manipulation of thermodynamic and kinetic conditions [111, 116, 117]. When using the GASAR process to fabricate porous metallic structures, it is important to address three key factors: the distinction in gas solubility amid the liquid and solid states, the velocity of directional solidification, and the shape of the solid–liquid interface [115, 116]. The solid–liquid interface morphology may change from planar or cellular under higher solidification velocity and temperature gradient conditions to columnar dendritic under lower ones [115, 119].

**Figure 4.**
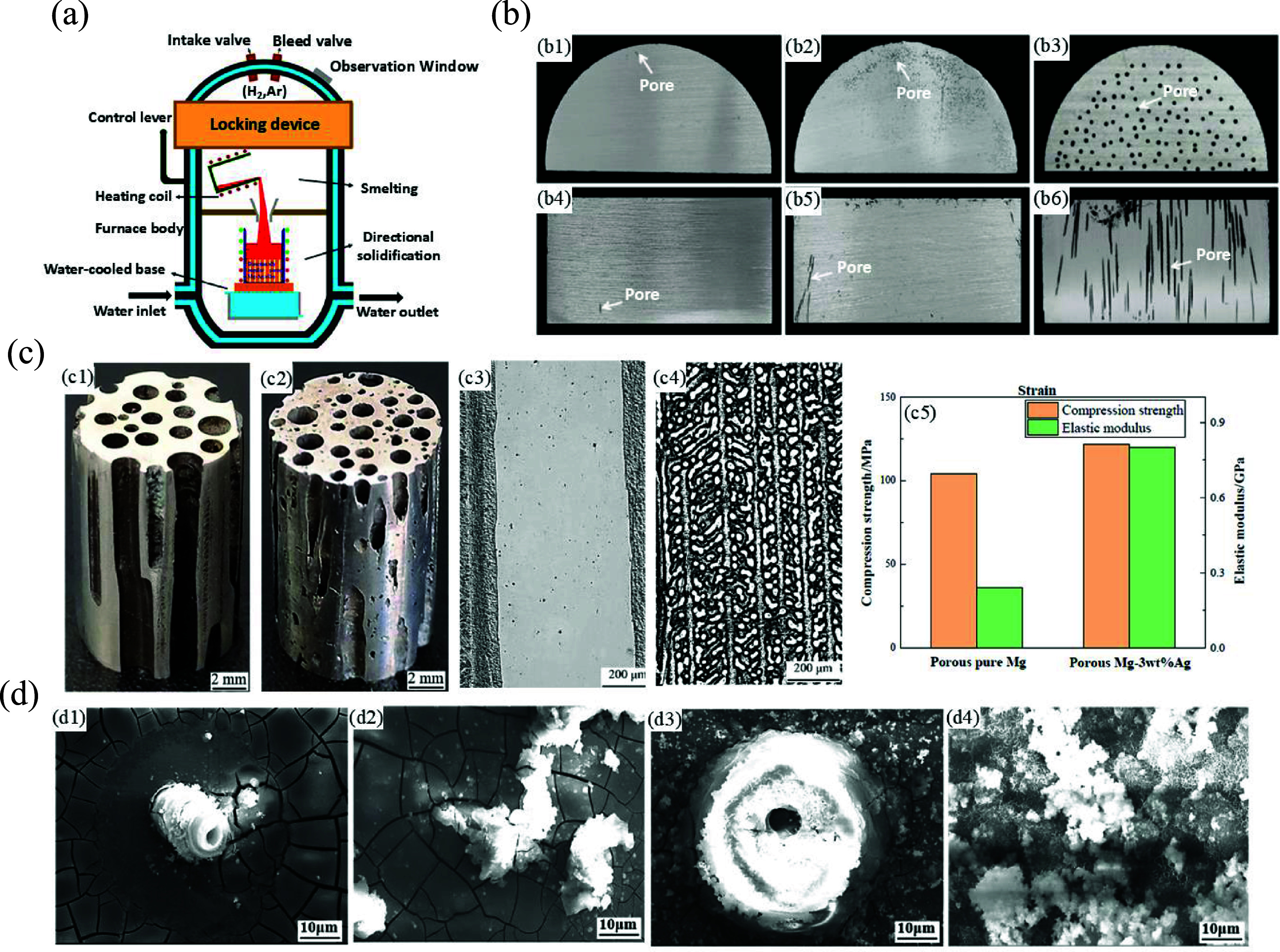
(a) Diagrammatic representation of the inner arrangement of the furnace body of the directional solidification device. (b) Differentiation of Mg–Ag eutectic alloy macroscopic pores with hydrogen pressure, (b1–b3): cross sections at 0.1, 0.2, and 0.6 MPa, respectively; (b4–b6): longitudinal sections at 0.1, 0.2, and 0.6 MPa, respectively. Reprinted from [125], © 2023 Elsevier Ltd All rights reserved. (c) The pictures (c1, c2) and the microstructures (c3, c4) of the porous pure Mg (c1, c3) and Mg-3 wt.% Ag alloy (c2, c4), and (c5) the compressive properties of the samples. (d) The surface morphologies of the porous pure Mg (d1, d2) and Mg-3 wt.% Ag alloy (d3, d4) of immersion in SBF at 37 ° C after 3 h (d1, d3) and 36 h (d2, d4). Reprinted from [128], © 2022 Elsevier B.V. All rights reserved.

Porous metallic structures composed of copper (Cu) [120, 121], aluminum (Al) [122], silver (Ag) [123, 124], and Mg [114, 118, 125–127] were fabricated using the GASAR process. Among various Mg-based alloys, the GASAR technique has been notably used to fabricate Mg–Ag scaffolds. In this regard, Li *et al* [125] inspected the impact of hydrogen pressure on the formation of macroscopic pores and the solidification structure of the porous Mg–Ag eutectic alloy utilizing the GASAR technique (figure 4(a)) [125]. The findings indicated that as the hydrogen pressure increases, the temperature gradient also increases at the front end of the solid/liquid interface, thereby enhancing the formation and expansion of pores, resulting in more uniform and regular directional growth of pores (figure 4(b)) [125]. Additionally, the increased temperature gradient results in a greater driving force for the development of the solidification structure, leading to the destabilization of the eutectic cells. Consequently, the eutectic structure transitions from a lamellar to a rod-like shape. In a similar work, Liu *et al* [128] used the GASAR technique to produce pure Mg (figure 4(c-c1, c3)) and Mg–3Ag (wt.%) alloy (figure 4(c-c2, c4)) scaffolds possessing well-structured pore arrangements for bone tissue engineering applications. In this investigation, the authors examined how the addition of Ag affected the mechanical characteristics and initial *in vitro* biodegradation behavior of the Mg–Ag alloy scaffolds. Ag addition substantially improved the CS and elastic modulus of the porous Mg–Ag alloy, as shown by mechanical evaluations (figure 4(c-c5)). Furthermore, when subjected to immersion trials in simulated body fluid (SBF) at 37 °C (figure 4(d-d4)) [128], it was shown that the Mg–3Ag alloy underwent corrosion at an increased rate. However, the inclusion of Ag aided in the formation of calcium and phosphorus salts, leading to improved osteoinductivity of the samples.

### 2.2. Pattern casting

Pattern casting methods are considered traditional methods for fabricating Mg-based scaffolds. Of the pattern casting methods, IC is the most common technique to produce Mg-based scaffolds [106, 107, 130]. IC, also known as precision casting or lost wax casting, involves encasing a wax model with refractory materials to create a disposable ceramic mold. IC is often preferred over other casting techniques due to the high level of detail and exceptional surface finishes achieved in the final products. This allows the IC technique to create structures with thin walls and intricate internal passages. These process characteristics can produce products that closely resemble the intended final form, resulting in significant cost savings in terms of materials, labor, and machining [131–133].

The IC process has some drawbacks, such as the possibility of the molten metal not fully penetrating all parts of the mold due to narrow pathways and the presence of air. One effective approach to address this issue is to use the VIC process [106, 134, 135]. Another strategy to improve melt penetration into narrow pathways is to use vibration during the pouring and solidification of the molten Mg-based alloy, which can be employed in both IC and VIC techniques [136, 137]. In the VIC method, the mold is prepared in the same way as IC, but the air is removed from the cavity before filling it with the molten Mg-based alloy. To prevent the liquid Mg-based alloy from oxidizing, melting and pouring the molten metal into the mold are carried out under vacuum conditions, typically using a VIM [134, 135]. In the case of Mg-based alloys, the reactions between the Mg-based melt and the mold can produce a non-desired oxide layer on the part surface. To resolve this issue, silica-free slurries with high stability may be used for creating disposable molds [138, 139]. Another technique involves using inhibitors, which are fluoride-based chemicals that react with the molten Mg to form a protective surface layer in the casting [138, 139]. Using this method, Herrero-Dorca *et al* [140] conducted tests on traditional SF_6_, alternative KBF_4_, and NaBF_4_, as well as environmentally friendly FK inhibitors. The study found that KBF_4_ is the optimal inhibitor for Mg during the IC [140, 141].

**Figure 5.**
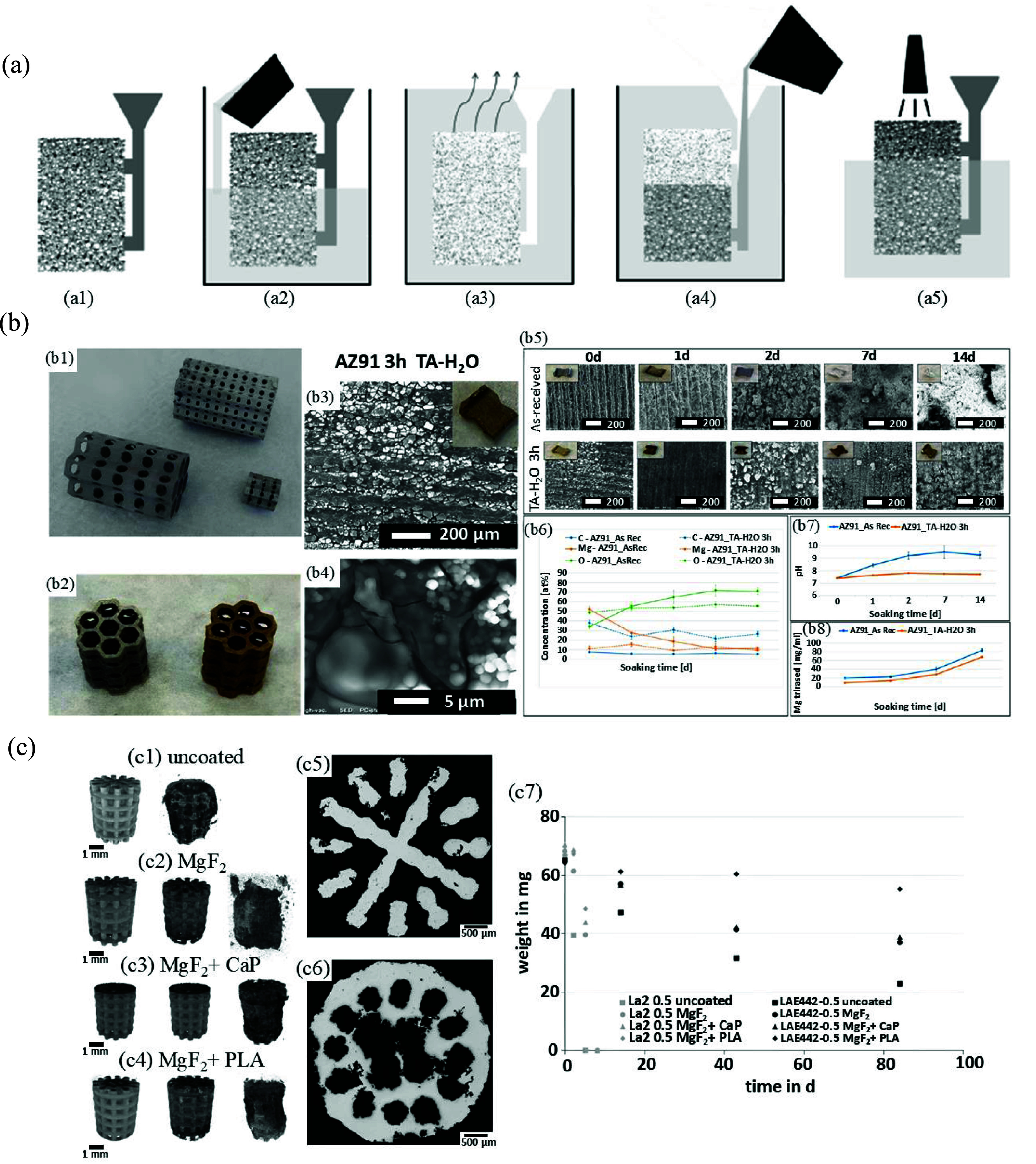
(a) Illustration of the investment casting procedure for metallic foams, including: (a1) preparation of the polymeric pattern with gating system, (a2) placing the polymeric pattern with gating system at the bottom of the mold and then pouring the mold with a ceramic-based material, (a3) drying and hardening the ceramic part and evaporating the polymeric pattern by heating the ceramic-polymer composite, (a4) foam casting by pouring the molten metal into the ceramic mold, and (a5) elimination of the ceramic mold and gating system. Reproduced from [148]. CC BY 4.0. (b) AZ91 porous structures (b1), images of small porous structures are shown before (on the left) and after (on the right) the application of the TA coating (b2), the SEM and macroscopic images of the AZ91 sample are shown after applying a TA coating using the dip coating method in a water solution for a duration of 3 h (b3, b4), the degradation tests yielded the following results: (b5) visual appearance and surface topography seen using SEM, (b6) surface chemical composition determined through EDS analysis, (b7) pH of PBS at various soaking times (*), and (b8) Mg concentration in PBS at various soaking times (*).(*) the study involved doing a solution refresh every 3 d. Reproduced from [142]. CC BY 4.0. (c) 3D tomographic images of the Mg–La2 scaffolds in (c1) uncoated and (c2–c4) coated conditions: virgin sample (left column), after 2 d (middle column) and after 5 d (right column) in SBF (every sample has a 0.5 mm pore size.), optical cross sections of Mg–La2 scaffolds with MgF_2_ + PLA coating after (c5) 2 d and (c6) 5 d in SBF, and (c7) weight of Mg–La2 and LAE442 scaffolds with various deterioration times (the average data from three specimens is represented by symbols, with the maximum weight variation for each set being less than 4 mg). Reproduced from [107]. CC BY 4.0.

The IC process consists of several steps, which are briefly reviewed as follows: the first step in IC is to design and produce the casting pattern. PU is a common polymer used to make the initial patterns in IC. AM techniques like FDM are considered innovative methods that have been widely used in various studies to fabricate these initial patterns using polymers or wax for casting porous metallic structures [107, 130, 133, 142, 143]. The basic porous patterns, primarily made of PU, are widely available in various volumes and shapes. The porosity of these patterns is mainly determined by the number of PPI and the diameter of the pores [144, 145]. Then, these patterns are attached to a gating system. The combination of pattern-gating systems is then covered with a refractory slurry made of ceramic. This can be done using different coating methods, such as dipping into a ceramic slurry (dip coating) or using CSC. After the refractory coating reaches the desired thickness, it undergoes a process of drying and hardening. Then, the molds undergo heat treatment, during which the polymeric patterns are incinerated to form a hollow structure that replicates the burned pattern precisely. Another technique to make the ceramic mold is to place the polymeric pattern with gating system at the bottom of the mold and then pour the mold with a ceramic-based material [146]. The ceramic-polymer composite is then heated to harden the ceramic part and evaporate the polymeric pattern. What remains is a ceramic-based mold with an internal hollow structure similar to the burned polymeric pattern. In IC, as the ceramic mold used in the IC process is destroyed during the process, a new casting pattern is necessary for each casting [132, 134, 135, 138, 147]. The next stage involves subjecting the produced casting pattern to heat. This process enhances the strength of the mold, removes any remaining wax or impurities, and evaporates the water content of the mold material. Subsequent to metal casting, the ceramic molds are eliminated, resulting in the desired porous Mg-based structure. Afterwards, the individual specimens are detached from their gating system using various methods such as sawing, cutting, burning, or cold breaking with liquid nitrogen, depending on the material and structural delicacy [131, 141]. A schematic illustration of the IC technique for fabricating porous metallic structures is shown in figure 5(a) [148].

Using an IC process and the evaporation of bare and wax-based slurry-thickened PU scaffold patterns with 20 and 10 PPI architectures, Kapłon *et al* [106] created high-porosity AZ91 Mg-based alloy scaffolds with an interconnected pore structure for temporary bioabsorbable bone implants. The porosity of scaffolds made from 20 PPI PU patterns ranged from 81% to 87%. On the other hand, scaffolds made from 10 PPI patterns had porosity between 91% and 92% in standard-coated PU patterns (like the 20 PPI samples) and 83%–86% in thick-coated PU patterns. Evaluation of their compatibility in SBF showed that the released metallic elements did not exceed the adult human daily requirement. The delay in the release of the alloy components could be attributed to the local passivation of the scaffold’s surface and the formation of a thin layer of apatite ceramics. Furthermore, two different PEO coatings were effectively applied to introduce diverse ceramic surface layers onto the scaffolds. The compact inner layer of these coatings is proposed to provide efficient protection against corrosion, while the porous external layer may be favorable for bone implant applications by offering a greater surface area for the growth of HA and enhancing the adherence rate [106].

Spriano *et al* [142] fabricated a honeycomb perforated structure pattern using PLA with the FDM technology and used it to produce AZ91 Mg-based alloy scaffolds through IC (figure 5(b-b1)). The produced samples were subsequently coated with TA (figure 5(b-b2)) using both water and PBS solutions through a dip coating process. This investigation showed that immersing samples in a TA/water solution for 3 h (figure 5(b-b3, b4)) resulted in a uniform coating on the entire three-dimensional (3D) surface. Degradation evaluation of samples, which was performed by immersing them in PBS for up to 14 d (figure 5(b, b5–b8)) [142], indicated the pH of PBS used in the degradation tests remained near the physiological pH for the coated samples but increased to 8.5 after 1 d and 9.5 after 7 d for the uncoated specimens. The TA coating functions as a barrier against dissolution by uniformly covering the Mg alloy surface, preventing it from interacting with the surrounding liquid medium. The results indicated that this coating shows promise in regulating the degradation of Mg-based alloys for biomedical purposes [142].

It is important to note that the use of TA has become significantly important as a useful chemical in the field of biomedical engineering [150–152]. TA exhibits anti-inflammatory, antibacterial, antiviral, antifungal, and anticancer properties. Within the area of biomaterial research, TA can be employed in producing hydrogels, thin films, and nanoparticles for wound healing, tissue regeneration, and drug delivery [150]. TA activity against many viruses, such as Influenza A, Papilloma, noroviruses, Herpes simplex type 1 and 2, and HIV, was also documented. In addition, TA exhibited antimicrobial action against both Gram-positive and Gram-negative bacteria, such as *S. aureus, E. coli, Streptococcus pyogenes, Enterococcus faecalis, P. aeruginosa*, Yersinia enterocolitica, and Listeria innocua [153].

Maeir *et al* [107] fabricated scaffolds using three different Mg-based alloys—Mg-La2, LAE442, and ZX61—using a combination of FDM and IC processes and examined the *in vitro* decomposition behavior of specimens under uncoated, MgF_2_ single-layer coated, MgF_2_ + CaP double-layer coated, and MgF_2_ + PLA double-layer coated conditions. Additionally, they investigated the *in vivo* degradation behavior of MgF_2_-coated samples with Mg–La2 and LAE442 substrates. The results showed that different coatings could improve the corrosion resistance of Mg–La2, but all the coated samples completely degraded after 8 d, which is relatively short for bio-applications (figure 5(c, c1–c4)). Uncoated Mg–La2 samples underwent complete and non-uniform degradation after 5 d of immersion in SBF (figure 5(c-c5, c6)). In comparison to LAE442, the degradation rate of Mg–La2 was significantly higher (figure 5(c-c7)) [107]. After 12 weeks, the uncoated LAE442 scaffolds still retained 35% of their original mass. The combination of MgF_2_ and PLA proved to be the most effective coating in reducing the degradation rate in both alloys. The correlation between the degradation rates of LAE442 and Mg–La2 in living organisms was consistent with the pattern observed in the *in vitro* studies, although at a much slower rate. The ZX61 alloy displayed a degradation rate similar to that of LAE442 in the initial stage, followed by a subsequent increase, which could be considered advantageous compared to LAE442 for bio-applications [107].

### 2.3. Techniques based on utilizing space holders

Fabrication techniques using space holders are commonly used to produce metallic scaffolds. These techniques can create scaffolds with either consistent porous structures or with varied pore size and/or porosity [154–156]. Different types of particles with varying chemical compositions, shapes, and sizes can be used as space holders, providing the advantage of creating metallic scaffolds with diverse specifications.

Space holders can be removed using techniques such as leaching [4, 157, 158], thermal decomposition [159–161], melting, and etching [149, 162, 163], depending on their properties. When space holders are leached, there is a risk of corrosion of metallic scaffolds. To address this, different materials (organic or inorganic) can be used in the solution to act as corrosion inhibitors. For example, potassium permanganate (KMnO_4_) can be used as a corrosion inhibitor in a NaCl solution for AZ31 Mg-based alloy [164].

**Figure 6.**
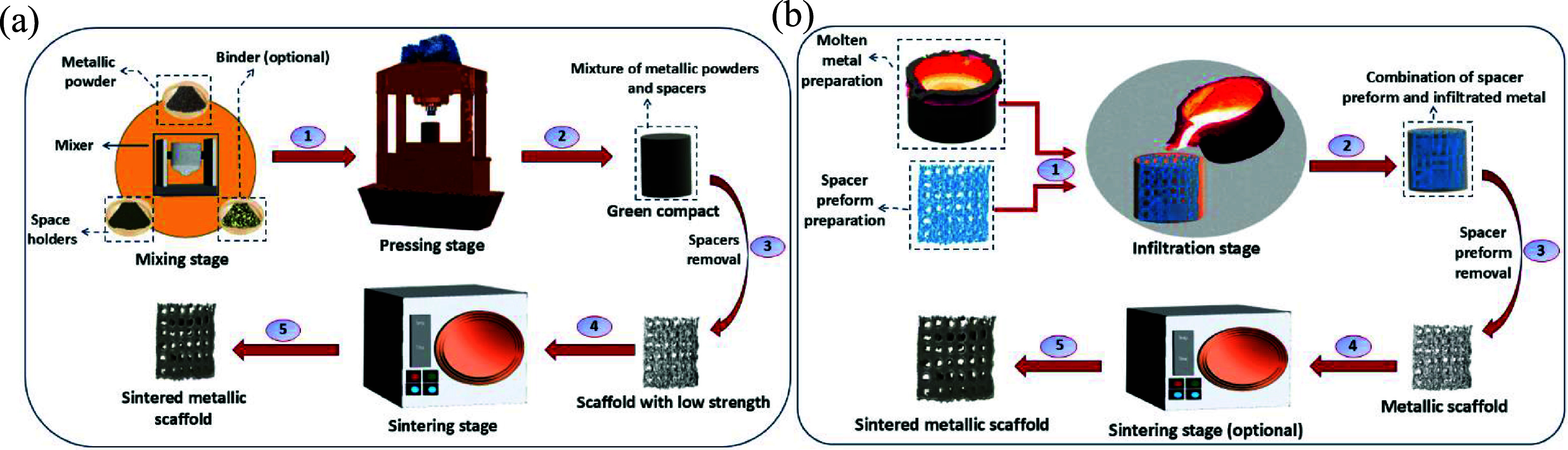
Schematic illustration of producing metallic scaffolds utilizing space holders through (a) P/M fabrication approach and (b) preform infiltration technique.

Generally, there are two main fabrication procedures for making metallic scaffolds with interconnected-pore structures using solid space holders:

• Powder metallurgy (P/M) technique: Metallic powders, space holders, and usually a binder are mixed and compressed under pressure at a high temperature to form a green compact. The space holders are then removed, and the structure is sintered to increase bonding strength between metallic powders (figure 6(a)). In some studies, the sintering process is done before the elimination of spacers [165–168].
• Preform infiltration technique: A porous preform of space holders is made, and then the target metallic melt is infiltrated into the preform using methods like hydraulic pressure, gas pressure, or a vacuum system. After solidification of the metallic melt, the space holders are removed, leaving behind the metallic scaffold with the same pore structure (figure 6(b)). Additional thermal treatments can be employed to increase the strength of the metallic scaffold [169–171].

The selection of a suitable spacer is crucial because it directly influences various structural parameters, such as the proportion of porosity and the shape of the pores. It also plays a key role in determining the process of removing the spacer, the sintering technology (if required), and the size and shape of the final scaffold. Ultimately, it also impacts the cost of the final fabricated metallic scaffold. Various materials have been used as spacers for fabricating porous metallic structures with interconnected pore geometry for both industrial and biomedical applications, such as sodium chloride (NaCl) [4, 172–174], magnesium sulfate (MgSO_4_) [175], carbamide (urea, (NH_2_)_2_CO) [61, 176, 177], saccharose (C_12_H_22_O_11_) [178–180], acrawax [181–183], ammonium bicarbonate (NH_4_HCO_3_) [184, 185], potassium carbonate (K_2_CO_3_) [186], PMMA [129, 187, 188], titanium (Ti) wire [162, 189], and camphene (C_10_H_16_) [109]. Properties, advantages, and disadvantages of the space holders that have been widely used to fabricate Mg-based scaffolds are reviewed in table 2.

**Table 2.** Illustration, advantages, and drawbacks of the most common space holders for fabricating Mg and Mg-based scaffolds.

Space holder/illustration	Advantages	Drawbacks	Remarks	Refrences
Sodium chloride (NaCl) 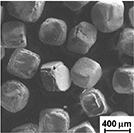 Reprinted from [4], Copyright © 2010 Elsevier B.V. All rights reserved.	• No toxicity in case of using for bio-applications, even if the space holders were not completely removed. • High melting temperature • Inexpensive and abundant material. • Rapid dissolution in water.	• If the space holders are not removed, Cl- ions can increase the scaffold’s corrosion rate. • The potential for the metal to interact with NaCl • Extended leaching times can cause Mg scaffold corrosion as a result of water interaction.	• Leaching is the most common technique for removing NaCl particles. • One of the most common space holders • Leaching increases with water temperature or NaCl particle dissolution time. • The melting point of the NaCl is 801 °C. • Sodium hydroxide solution is effective for extracting sodium chloride particles through leaching	[4, 169, 174, 190–194]

Carbamide (urea, (NH_2_)_2_CO) 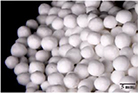 Reprinted from [61], Copyright © 2010 Elsevier Ltd All rights reserved.	• Inexpensive and abundant material. • Leachable in water, NaOH solution, and ethanol. • Can be removed by thermal decomposition. • Carbamide low decomposition temperature is advantageous for preserving the metallic scaffold structure.	• Some metals may experience undesired reactions or phase changes during the carbamide removal stage by thermal decomposition. • NH_3_ and CO_2_ gases, which are produced due to the thermal decomposition of carbamides, can remain trapped within the scaffold structure, affecting its mechanical properties and corrosion behavior.	• Due to the thermal decomposition, carbamide decomposes into ammonia gas (NH_3_) and carbon dioxide (CO_2_). • Although leaching technique can be used for carbamide removal, thermal decomposition is the most common method for removing carbamide particles. • The biocompatibility of carbamide-derived gases must be evaluated since ammonia and carbon dioxide could potentially affect tissue response.	[61, 158, 167, 176, 177]

Ammonium bicarbonate (NH_4_HCO_3_) 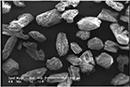 Reprinted from [85], Copyright © 2015 Published by Elsevier Ltd.	• The capacity to entirely disintegrate at comparatively low temperatures compared to Mg melting point.	• Obtaining accurate regulation of pore size and distribution might be difficult.	• According to some studies, keeping at 175 ° C for 2 h is adequate for removing NH_4_HCO_3_ particles through thermal decomposition. • Ammonia (NH_3_), carbon dioxide (CO_2_), and water (H_2_O) are the products of ammonium bicarbonate thermal decomposition.	[85, 195, 196]

Camphene (C_10_H_16_)	• Sublimation of camphene can be done by utilizing a medium vacuum at room temperature. • Camphene can also be removed by thermal decomposition at low temperatures compared to the Mg melting point.	• Applying high temperatures during the mixing of camphene and the target metallic powder can lead to undesired sublimation of camphene particles before compaction. • When using camphene as spacers, applying infiltration techniques for fabricating Mg scaffolds is not possible due to the high temperature of molten Mg.	• Camphene is insoluble in water but soluble in organic solvents like ethanol, diethyl ether, and chloroform.	[109, 197, 198]
Polymethylmethacrylate (PMMA) 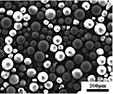 Reprinted from [129], Copyright © 2015 Elsevier B.V. All rights reserved.	• PMMA in an inexpensive and abundant material • Accurate control over the porosity and pore size of the resulting metallic scaffold. • PMMA is biocompatible. • PMMA can be easily removed by thermal decomposition.	• The decomposition of PMMA can leave behind residual carbon in the metallic scaffold. This carbon residue may alter the material properties.	• The use of PMMA as a space holder ensures uniform and well-defined pores. • PMMA removal by thermal decomposition can be done throughout the sintering stage. • Thermal decomposition of PMMA can lead to releasing volatile byproducts, affecting the overall quality of the scaffold. • PMMA decomposes at 360 °C without oxygen because to its low melting point and thermal stability.	[129, 187, 188]

Ti wire 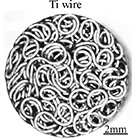 Reprinted from [149], © 2016 Elsevier B.V. All rights reserved.	• Capable of fabricating Mg scaffolds with controlled porosity percentage and 3D pore structures and morphology. • The linked pores formed by the Ti wire space holder (TWSH) method facilitate bone development into the scaffold. This is essential for achieving successful integration and ensuring stability. • Formation of MgF_2_ coating on the surface of Mg-based scaffold during Ti wire etching, which significantly enhances the corrosion resistance.	• Mg and Ti have distinct chemical compositions. Chemical interactions between the two materials may occur when Ti wires are used as spacers inside a Mg matrix. The general integrity of the porous structure may be impacted by these interactions, which could result in corrosion. • Ti and Mg can form a galvanic pair, with Ti being the more noble material that corrodes the less noble material, Mg. This can decrease the corrosion resistance of porous Mg. • Extracting the Ti wires post-sintering or consolidation may provide difficulties. The wires can get embedded in the Mg matrix, which can make it challenging to remove them without harming the scaffolds’ integrity.	• The biological reaction at the implant site may be impacted by leftover Ti particles or ions within the Mg matrix, notwithstanding Ti overall biocompatibility. • Hydrofluoric acid (HF) solution is mainly used for etching (removing) Ti wires. • The porous structure can be filled with bioactive chemicals or medications to improve tissue regeneration or prevent infections. • The TWSH technique surpasses AM procedures due to its superior precision. Unlike AM techniques, which are limited in their ability to synthesize Mg-based scaffolds with primary holes greater than 500 *μ*m.	[149, 162, 163]

Witte and associates [169] manufactured AZ91D Mg-based scaffolds with pore sizes ranging from 10 to 1000 *μ*m and porosity between 72% and 76%, using an infiltration technique and NaCl spacers. Afterwards, scaffolds were inserted into the distal femur condyle of rabbits and contrasted with autologous bone (as a control), which was transplanted into the contralateral condyle in 3- and 6 month follow-up groups. The *in vivo* results demonstrated that the scaffolds degraded over 3 months, removing most of the initial AZ91D Mg-based scaffold. It was also indicated that the surgical site was surrounded with a fibrous capsule. Histological analysis revealed that AZ91D Mg-based scaffolds did not damage adjacent tissues. This investigation also found that even rapidly degrading AZ91D Mg-based scaffolds were biocompatible and provoked an appropriate inflammatory host response *in vivo* [169].

Wang *et al* [170] produced Mg scaffolds with a cell wall thickness ranging from 0.2 to 0.6 mm. They achieved this by vacuum-assisted infiltration of molten Mg into a preform of spherical salt particles, followed by the removal of the salt particles in water containing 0.001 M NaOH. Analysis of the scaffolds’ microstructure revealed two main types of pores: macropores (1.5 mm in diameter) formed due to the dissolution of salt particles, and smaller pores (0.6–0.9 mm in diameter) located mainly at the cell center. The mechanical assessment showed that the CYS of the Mg scaffolds increased as the porosity decreased. The CYS of Mg scaffolds with porosities ranging from 54% to 70% ranged from 3.6 to 8.7 MPa, with corresponding elastic moduli ranging from 21 to 50 GPa. These mechanical parameters are consistent with those typically observed in cancellous bone [170]. Table 3 provides the mechanical parameters of Mg-based alloy scaffolds manufactured using various fabrication techniques, along with corresponding notes regarding their mechanical properties.

**Table 3.** Mechanical properties, enhancement mechanism, and mechanical remarks of the scaffolds made of Mg-based alloys.

Fabrication technique	Materials	Porosity (%)/pore diameter	Compressive/tensile properties (MPa)	Energy absorption capability (MJ.m^−3^)	Young’s modulus (GPa)	Enhancement/remark	References
Space holder (melt infiltration)	Mg scaffolds	67%	CYS = 2.5	EAC = 5.5	0.72	The sample with reduced porosity percentage and pore diameter had superior mechanical properties, such as a higher Young’s modulus, CYS, and elevated EAC.	[171]
		PS = 1 mm					
		73%	CYS = 1.9	EAC = 4.6	0.69		
		PS = 1.4 mm					
		76%	CYS = 1.5	EAC = 3.1	0.65	The mechanical properties determined in this study meet the necessary requirements for scaffold materials, energy absorbers, filters, catalyst supports, and CO_2_ capture media.	
		PS = 1.7 mm					
		78%	CYS = 1.2	EAC = 1.9	0.61		
		PS = 2 mm					

Space holder (P/M technique)	Mg–1 wt.% Ca–1 wt.% Mn–6 wt.% Zn	60%	UCS = 4.0 ± 0.2	—	—	The inclusion of just 0.5 wt.% of Ag improves the UCS. This improvement can be due to the existence of the fine precipitate phase of Mg−Ag, the refinement of the grain structure, and the presence of a weak basal texture.The increase in Ag concentrations (2.0 wt.%) resulted in a further enhancement of the UCS due to the process of grain refining, which in turn hindered both grain boundary sliding and dislocation motion.	[167]
		PS = 600–800 *μ*m					
	Mg–1 wt.% Ca–1 wt.% Mn–6 wt.% Zn–0.5 wt.% Ag	60%	UCS = 4.3 ± 0.2	−	—		
		PS = 600–800 *μ*m					
	Mg–1 wt.% Ca–1 wt.% Mn–6 wt.% Zn–2.0 wt.% Ag	60%	UCS = 4.8 ± 0.2	—	—		
		PS = 600–800 *μ*m					

Space holder (P/M technique)	Mg scaffold	50%PS = 200–500 *μ*m	CS = 2.33	—	0.35	Pores ranging from 400–500 *μ*m occur more frequently than those sized 200–400 *μ*m.	[168]
						The average compressive densification strain of the Mg scaffolds was around 50%, which makes them a good candidate for energy absorbent applications.	

Space holder (P/M technique)	Mg scaffold	55%	CS = 15 ± 3	—	4 ± 2	The CS, compressive flexure strength (CFS), and Young’s modulus of the porous Mg specimens decline as the porosity rises.	[199]
		PS = 200–400 *μ*m	CFS = 14 ± 3				
Space holder (P/M technique)	Mg scaffold	35%	CPS = 17	—	1.8	The Young’s modulus and compressive peak stress (CPS) of Mg scaffolds increase with a decrease in porosity and pore size.	[200]
		MPS = 250 *μ*m					
		45%	CPS = 16	—	1.3		
		MPS = 73 *μ*m					

Space holder (P/M technique)	Pure Mg	63%–65%	CS = 3.2	—	—	Due to the minimal impact of tetracycline on the quantity and structure of porosity, its incorporation into the scaffolds does not influence the mechanical strength of the scaffolds.	[201]
	Mg–6 wt.%Zn	63%–65%	CS = 4.8	—	—	Zn can serve as a component to enhance the microstructure of the Mg matrix and improve Mg mechanical stability. The CS of both pure Mg and tetracycline-loaded Mg–Zn scaffolds decreased after being immersed in a SBF solution for 72 h.	

Space holder (P/M technique)	Mg–1 wt.%Ca–6 wt.% Zn	Around 60%	CS = 3.4 ± 0.2	—	—	Zeolite (Zeo) acts as a rigid filler in a Mg matrix, hence enhancing the mechanical strength and increasing the CS of Mg-based scaffolds. The presence of Zeo at the surfaces of grain boundaries may also be responsible for this phenomenon, as it acts as an obstacle to the movement of dislocations and leads to an increase in dislocation density due to the mismatch in strain between the Zeo and the Mg-based matrix.	[202]
		PS = 600–800 *μ*m					
	Mg–1 wt.%Ca–6 wt.% Zn–3 wt.%Zeo	Around 60%	CS = 4.3 ± 0.3	—	—		
		PS = 600–800 *μ*m					
	Mg–1 wt.%Ca–6 wt.% Zn–5 wt.%Zeo	Around 60%	CS = 4.9 ± 0.3	—	—		
		PS = 600–800 *μ*m					
	Mg–1 wt.%Ca–6 wt.% Zn–7 wt.%Zeo	Around 60%	CS = 5.3 ± 0.4	—	—		
		PS = 600–800 *μ*m					

Space holder (P/M technique)	Mg scaffold coated 1 and 3 time by dip-coating in 10 g bioactive glass(BG)/100 mL PCL solution after immersion in SBF for 144 h	35%–40%	CS = 10 (1 time)	—	—	The CS of all the groups prior to immersion exhibited a comparable value, specifically 52 MPa. By incorporating PCL-BG coating, the degradation resistance of Mg scaffolds is enhanced, resulting in improved mechanical integrity of the samples when immersed in the SBF.	[196]
			CS = 17 (3 times)				
Space holder (P/M technique)	Mg scaffold	35%–40%	CS = 44	—	—	The CS of the Mg scaffold, which has a porosity of 35%–40%, is 32% of the CS of solid Mg with the same shape and diameter. Upon comparing the mechanical strength of sintered scaffolds with unsintered ones, it was found that sintering resulted in a tenfold boost in CS.	[203]
	Mg scaffold coated with PCL-BG/Gel-BG	35%–40%	CS = 44	—	—		
	Mg scaffold coated with PCL-BG/Gel-BG after 10 d immersion in SBF	35%–40%	CS = ∼12	—	—		

TWSH (preform infiltration)	Mg scaffold coated with MgF_2_	43.2%	CYS = 6.2	—	1.0	The mechanical characteristics of the porous Mg should be impacted by the MgF_2_ coating on the Mg surface, but this impact is minimal. This is due to the fact that pore shape and porosity have a significant influence on compressive mechanical characteristics.	[204]
		PS = 0.27 mm					
		51%	CYS = 4.6	—	0.6		
		PS = 0.27 mm					
		54.2%	CYS = 4.3	—	0.5		
		PS = 0.27 mm					

TWSH (preform infiltration)	Mg scaffold coated with MgF_2_	30%	CS = 83 ± 8	23 ± 2	—	High CS and specific strength, excellent EAC per mass, and excellent damping capacity were all displayed by the manufactured samples.	[149]
		PS = 0.27 mm					
		40%	CS = 74 ± 7	20 ± 2	—		
		PS = 0.27 mm					
		50%	CS = 59 ± 5	18 ± 2	—		
		PS = 0.27 mm					
		30%	CS = 121 ± 10	27 ± 2	—		
		PS = 0.1 mm					
		30%	CS = 71 ± 6	18 ± 2	—		
		PS = 0.4 mm					

TWSH (preform infiltration)	Mg scaffold coated with MgF_2_	55 ± 3%	CS = 41 ± 2	—	2.18 ± 0.06	The CS of the scaffolds fell between 0.2 and 80 MPa, which is the typical range for human cancellous bone.By altering the pore orientation, the mechanical properties could also be controlled without compromising the necessary porous structure.	[205]
		PS = 250 *μ*m					
		54 ± 3%	CS = 46 ± 4	—	2.37 ± 0.09		
		PS = 400 *μ*m					
AM (BJ)	Mg–Zn/5 Wt.% *β*-TCP	48 ± 11%	CYS = 24 ± 13	—	432 ± 103 MPa	The CYS of the Mg–Zn/5TCP and Mg–Zn/10TCP composite scaffolds were almost twice as high as the CYS of the Mg-Zn scaffolds. During the 28 day period of biodegradation, the mechanical characteristics of the composite scaffolds varied, but the CS and elastic moduli consistently fell within the spectrum of the mechanical properties of cancellous bone.	[206]
		PS = 360 *μ*m	CS = 41 ± 18				
	Mg–Zn/10 Wt.% *β*-TCP	49 ± 4%	CYS = 31 ± 2	—	585 ± 118 MPa		
		PS = 360 *μ*m	CS = 39 ± 23				
	Mg–Zn/10 Wt.% *β*-TCP	55 ± 18%	CYS = 23.5 ± 0.6	—	456 ± 54 MPa		
		PS = 360 *μ*m	CS = 30 ± 3				

**Figure 7.**
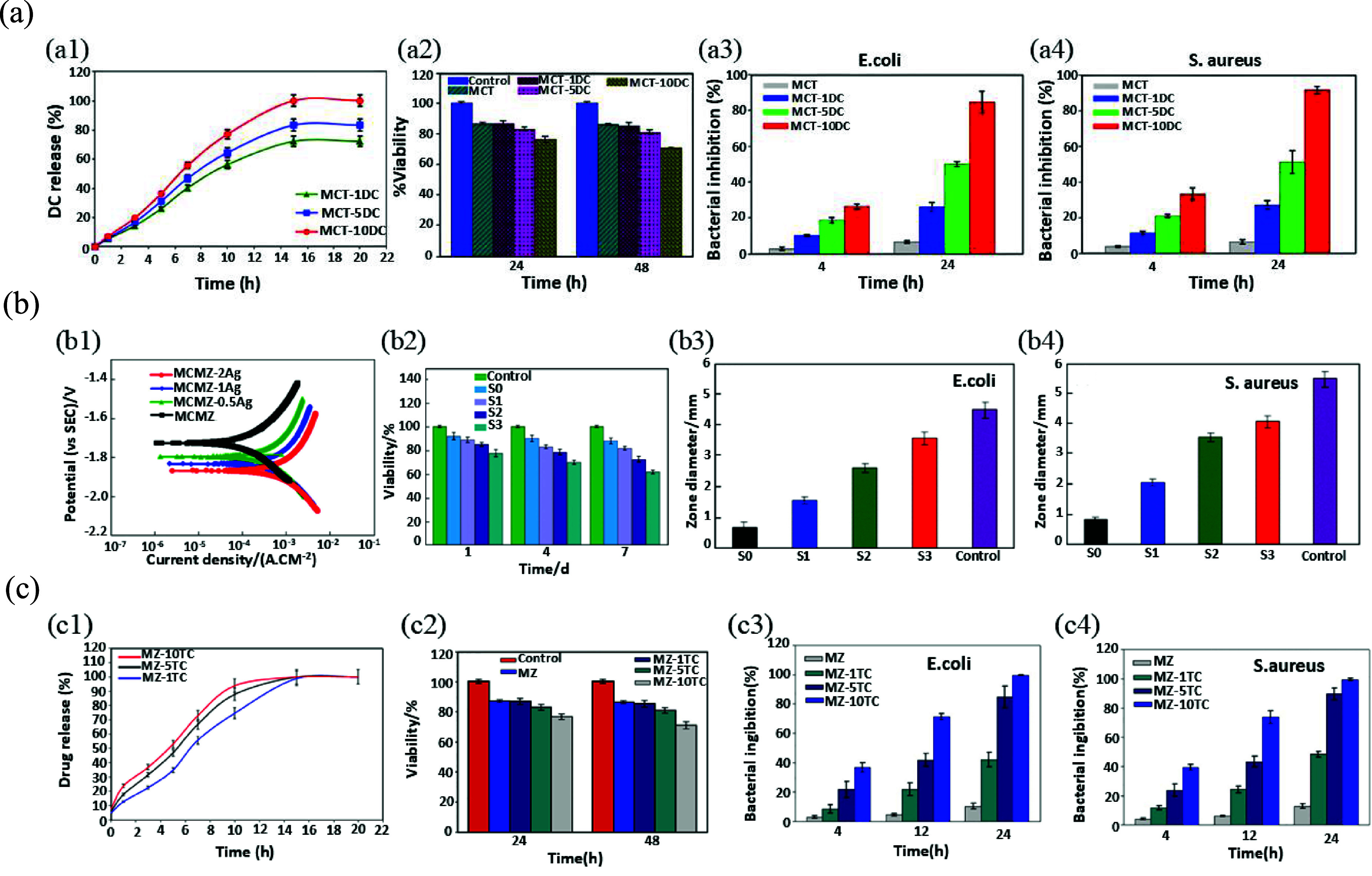
(a) (a1) drug release patterns of MCT-DC composite scaffolds with different DC amounts, (a2) viability of MG63 osteoblast cells cultured for 24 and 48 h on MCT-DC scaffolds, and the proportion of bacterial suppression of the MCT-DC scaffolds at different periods of incubation against (a3) *E. coli* and (a4) *S. aureus* bacteria. Reprinted from [208], © 2017 Published by Elsevier Ltd. (b) (b1) potentiodynamic polarization of MCMZ-*x*Ag scaffolds, (b2) viability of MG63 cells cultured on MCMZ−*x*Ag scaffolds for various periods, and values of growth-inhibition zones against *E. coli* (b3) and *S. aureus* (b4) after 24 h. (S0: MCMZ; S1: MCMZ-0.5Ag; S2: MCMZ-1Ag; S3: MCMZ-2Ag). Reprinted from [167], Copyright © 2019 The Nonferrous Metals Society of China. Published by Elsevier Ltd All rights reserved. (c) (c1) Drug release patterns of MZ-TC composite scaffolds with different amounts of TC, (c2) Viability of MG63 osteoblast cells cultured for 24 and 48 h on MZ-TC scaffolds, and the percentage of bacterial inhibition against (c3) *E. coli* and (c4) *S. aureus* of the MZ-TC scaffolds at different periods of incubation. Reprinted from [201], © 2019 Published by Elsevier B.V.

Hao *et al* [207] used carbamide particles as spacers to produce porous Mg with an interconnected pore structure using the P/M technique. They investigated key manufacturing parameters and found that optimal results were achieved at compacting pressures between 200–300 MPa and sintering temperatures of 610 °C–630 °C. Structural evaluation showed that the scaffold’s pores closely resembled the carbamide particles’ dimensions. In a comparable study, Bakhsheshi-Rad *et al* [208] used the same space holders and fabrication technique in order to fabricate MCT composite scaffolds containing 65%–67% interconnected porosity loaded with different concentrations of DC. The drug release profiles (figure 7(a-a1)) of samples indicated that both immediate and prolonged drug release were achieved by DC-loading MCT scaffolds and also demonstrated that the medication discharge rate heightened with rising DC content. Evaluation of the manufactured scaffolds’ cytotoxicity on MG63 cells (figure 7(a-a2)) showed the absence of toxicity in specimens with low concentrations of DC. Conversely, scaffolds containing a high concentration of DC showed some toxicity. All MCT-DC scaffolds in this investigation showed strong antibacterial properties against *S. aureus* and *E. coli* bacteria. The antibacterial effectiveness of the scaffolds improved as the concentration of DC rose (figure 7(a-a3, a4)) [208]. In another investigation by Bakhsheshi-Rad *et al* [167], the combination of carbamide particles and the P/M technique was used in order to fabricate Mg-based composite scaffolds containing various silver (Ag) concentrations, denoted by Mg–Ca–Mn–Zn–*x*Ag (MCMZ-*x*Ag) (where *x* is the silver concentration), in order to achieve scaffolds with enhanced antibacterial activities. The study found that increased silver concentrations not only resulted in decreased corrosion resistance of scaffolds (figure 7(b-b1)), but also caused higher cytotoxicity and lowered viability of MG63 cells (figure 7(b-b2)). However, these higher silver amounts enhanced the antibacterial effectiveness of MCMZ−Ag scaffolds (figure 7(b-b3, b4)) against both *E. coli* and *S. aureus*. The findings of this research demonstrated that 60% porosity and 600–800 *μ*m pore size in porous 0.5 wt.% Ag-MCMZ scaffolds provide the essential requirements for use in bone tissue engineering [167].

In a similar research, Dayaghi *et al* [201] utilized carbamide particles as spacers to manufacture Mg–Zn (MZ) scaffolds using the P/M technique. They used various tetracycline (TC) concentrations (MZ-*x*TC, where *x* = 1, 5, and 10%). The addition of TC did not impact the porosity of the scaffolds, as confirmed by microstructural evaluation. Both MZ and MZ-*x*TC (*x* = 1, 5, and 10%) maintained the same porosity with pore diameters ranging between 600 and 800 *μ*m and an interconnected pore structure. The MZ scaffolds displayed better corrosion resistance and CS compared to pure Mg scaffolds. Moreover, the incorporation of TC had little effect on the corrosion and mechanical properties of the scaffolds. Evaluation of drug release indicated that MZ-*x*TC scaffolds exhibited rapid drug release, followed by more consistent release patterns (figure 7(c-c1)). In SBF, bioactivity testing revealed that MZ-*x*TC scaffolds can form HA layers. According to the MTT assay on Mg63 cells, MZ scaffolds with 1%–5% TC better preserved cell viability (figure 7(c-c2)), while MZ-10TC showed some toxicity. Furthermore, antibacterial activity tests indicated that scaffolds with higher TC concentrations were more resistant to *E. coli* (figure 7(c-c3)) and *S. aureus* (figure 7(c-c4)) bacteria than MZ scaffolds without TC [201].

Further, Saheban *et al* [202] utilized carbamide spacers and a P/M fabrication process to fabricate Mg/zeolite (Zeo) scaffolds. Then, they added silver at 0.5 and 1 wt.% to the Mg-Zeo composite powder. These scaffolds had a pore diameter ranging from 600 to 800 *μ*m and a porosity level of approximately 60%. The analysis of corrosion behavior indicated that the inclusion of Zeo within Mg composite scaffolds leads to increased resistance to corrosion compared to Mg scaffolds without Zeo. Additionally, a positive correlation between the amount of Zeo and both cell attachment and proliferation was recorded through an MTT assay conducted on MG63 cells. Furthermore, the antibacterial activity of Mg/Zeo–Ag composite scaffolds suggested that the inclusion of silver into Mg-7 wt.% Zeo composite scaffolds could provide long-term antibacterial protection, making it a viable option for preventing bone infections [202].

**Figure 8.**
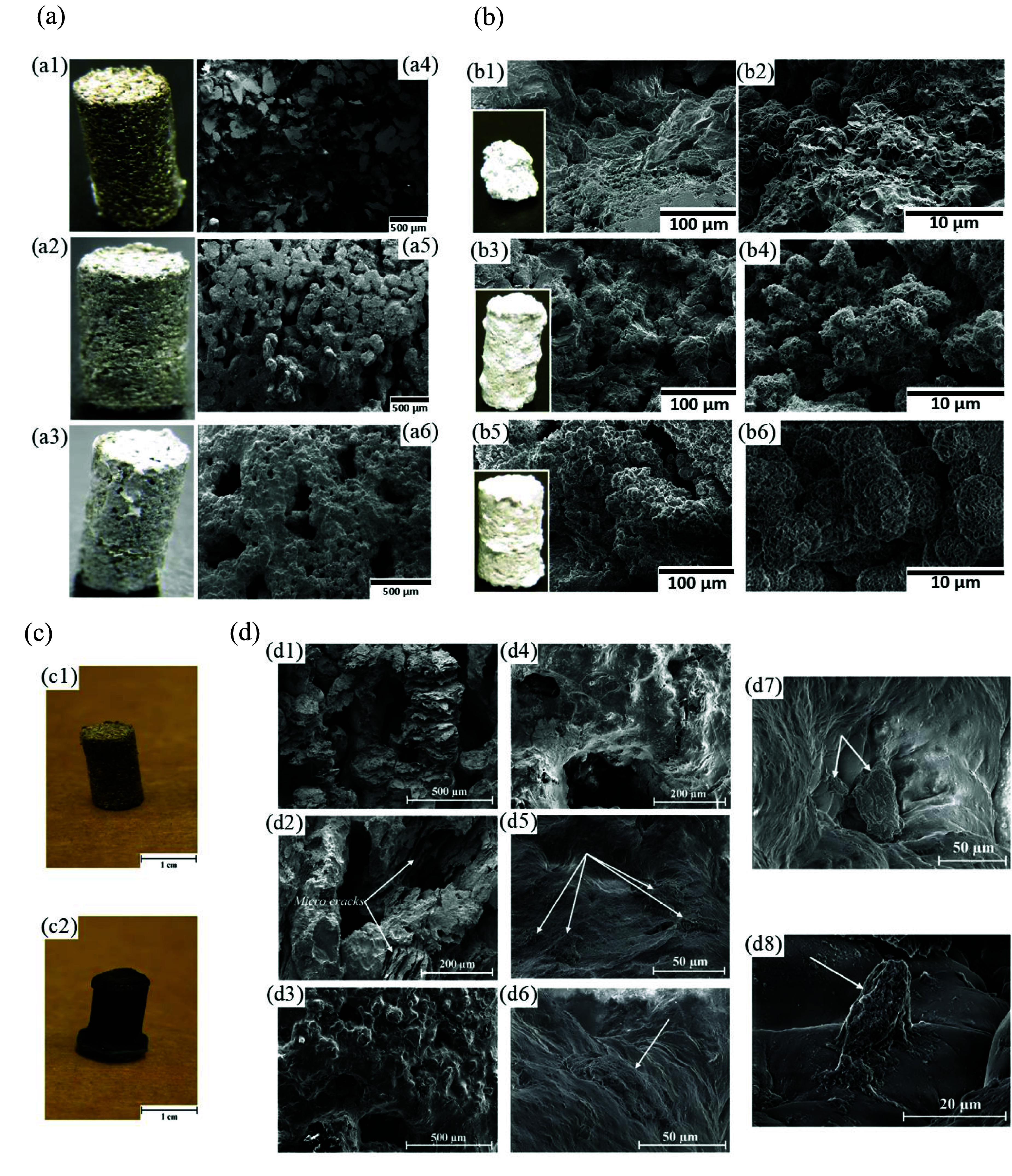
(a) Images of (a1) Mg scaffold, (a2) Mg scaffold/1PCL-BG, and (a3) Mg scaffold/3PCL-BG, SEM micrographs of (a4) Mg scaffold, (a5) Mg scaffold/1PCL-BG, and (a6) Mg scaffold/3PCL-BG. (b) (b1, b2) SEM micrographs and images of uncoated Mg scaffold, (b3, b4) Mg scaffold/1PCL-BG, and (b5, b6) Mg scaffold/3PCL-BG specimens immersed in the SBF after 48 h. Reprinted from [196], Published by Elsevier B.V. (c) Pictures of (c1) produced uncoated Mg scaffold, and (c2) Mg scaffold coated by 3PCL-BG/Gel-BG. (d) Deterioration morphology of (d1, d2) the bare Mg scaffold and (d3, d4) Mg scaffold coated by 3PCL-BG/Gel-BG (The pictures show the scaffolds’ architecture, which removes degradation products after a 48 hour immersing at SBF), and (d5–d8) SEM photomicrographs of the cells (human osteosarcoma cell line Saos-2) cultured for 3 d on the surfaces of the Mg scaffold coated by PCL-BG/Gel-BG at various locations that clearly show the attached cells on the surfaces (arrows). Reprinted from [203], © 2016 Elsevier B.V. All rights reserved.

As previously mentioned, ammonium bicarbonate, also referred to as carbonate hydrogen ammonium (NH_4_HCO_3_), is another common spacer. In this regard, Yazdimamaghani *et al* [196] used 150–300 *μ*m carbonate hydrogen ammonium powder as spacers to fabricate Mg scaffolds through the P/M technique and subsequently coated samples with PCL/bioactive glass (BG), using the dip-coating method. One and three layers of coating (1 PCL-BG and 3 PCL-BG, respectively) were utilized on the Mg scaffolds to assess the impact of the quantity of coating layers. Optical images and SEM micrographs of samples are shown in figure 8(a) [196]. The samples’ porosity ranged from 35 to 40 vol.%. Immersion of samples in SBF (figure 8(b)) revealed that the 1PCL-BG coated Mg scaffolds displayed substantially improved bioactivity, delayed degradation, and improved mechanical stability compared to Mg scaffolds without coating [196]. Additionally, it was demonstrated that a three-fold rise in PCL-BG coating concentration significantly enhanced the scaffolds’ mechanical stability, bioactivity, and resistance to degradation [196]. A related study [203] described Mg scaffolds in the previous investigation, which were coated with three layers of PCL/BG (3PCL/BG) through dip-coating, subsequently coated with a hydrogel/ceramic layer consisting of gelatin (Gel)/BG by freeze-drying technique. Figure 8(c) [203] shows the images of the uncoated Mg scaffold and Mg scaffold coated by 3PCL-BG/Gel-GB. In this investigation, evaluation of the degradation morphology of the scaffolds (figure 8(d, d1–d4)), after removing the degradation substances from the specimens, indicated that not only applying 3PCL-BG/Gel-BG coating strengthened Mg scaffolds’ resilience to degradation by sealing their surface but also increased the mechanical stability of specimens when immersed in the SBF compared to uncoated Mg scaffold. Cytocompatibility evaluation of scaffolds against the human osteosarcoma cell line Saos-2 revealed an acceptable cell viability and growth on the Mg scaffold coated by 3PCL-BG/Gel-BG (figure 8(d, d5–d8)) [203], while cells could not adhere to the bare Mg scaffolds.

Camphane is a less common substance used as a space holder for manufacturing porous Mg structures compared to other spacers such as sodium chloride or carbamide. Zou and Li [109] utilized camphene as spacers to produce Mg scaffolds with porosities ranging from 28% to 62% using the P/M method. The sublimation technique was utilized in order to eliminate camphane spacers in this investigation. It was indicated that the synthetic porous Mg samples exhibited high purity with minimal traces of MgO [109].

In the case of utilizing PMMA as spacers, Bi *et al* [129] produced porous Mg structures using the P/M technique, employing spherical PMMA with varying concentrations (up to 30 wt.%) as the spacer material. PMMA spacers were removed through thermal decomposition. The density of porous Mg decreased from 1.72 to 1.05 g cm^−3^ as the PMMA content increased from 0% to 30%, resulting in a corresponding increase in porosity up to 40%. Furthermore, evaluation of the mechanical properties indicated that the UCS varied from 25 to 170 MPa and increased as porosity decreased [129]. In a similar study, Tan *et al* [187] used the P/M technique and spherical PMMA spacers to manufacture porous Mg. The study found that the density of porous Mg structures increased while the porosity decreased as the sintering temperature increased. Moreover, mechanical evaluation of porous Mg specimens indicated that the CYS of fabricated specimens ranged from 20 to 23 MPa and increased as the PMMA particle size and porosity decreased [187].

Mg-based scaffolds can also be manufactured using a TWSH, following the same procedure as other space holders, employing either P/M or preform infiltration methods. To achieve the desired 3D porosity structure, a Ti wire mesh is intricately woven to serve as a spacer [209, 210]. The primary method for removing (etching) Ti wires and manufacturing Mg-based scaffolds is to immerse the fabricated Mg/Ti wire composites, obtained using P/M [162, 189] or preform infiltration [149, 204, 205] procedure, into a solution of HF [211]. An advantage of utilizing TWSH is generating an MgF_2_ layer on the surface of an Mg-based scaffold throughout the etching step of Ti wires [205]. Not only does this coating improve the resistance to degradation of Mg-based scaffolds [81, 212, 213], but several studies reported that this coating can enhance cytocompatibility, osteogenic activity, superior osteoconductive, and improved osteoinductive properties [81, 214].

**Figure 9.**
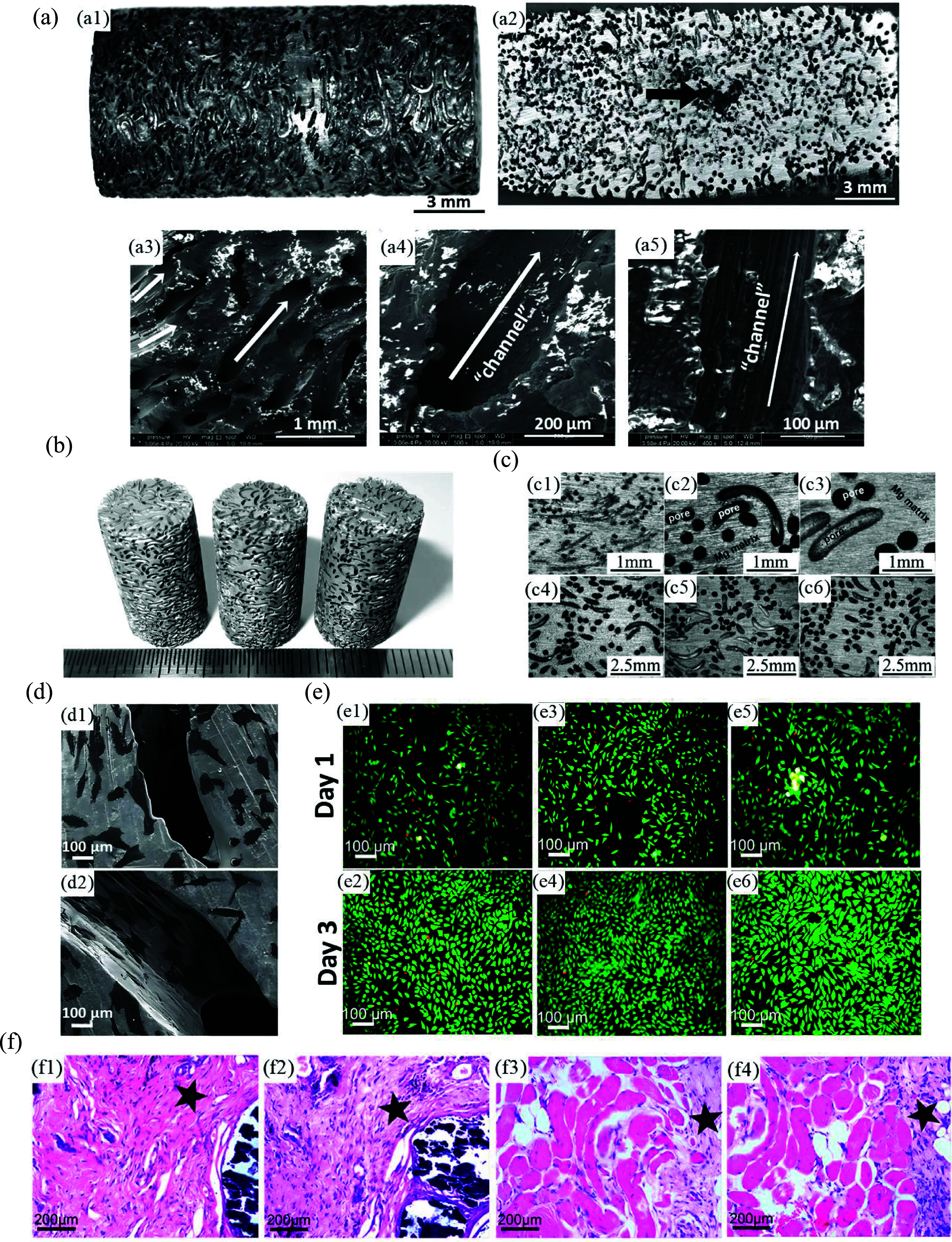
(a) (a1) Macroscopic view of the porous Mg as prepared, (a2) the section view (The black arrow indicates the cavity-like pore located at the center of porous Mg), and (a3–a5) the SEM micrographs of the section. Reprinted from [204], Copyright © 2014 Elsevier B.V. All rights reserved. (b) Macrographs of cylindrical porous Mg samples with 50% porosity and pore diameter of 0.27 mm. (c) Morphologies of the as-prepared porous Mg samples: (c1–c3) 30% porosity; pore diameter = 0.10 mm, 0.27 mm and 0.40 mm, respectively, and (c4–c6) 0.27 mm pore diameter; porosity = 30%, 40%, and 50%, respectively. Reprinted from [149], © 2016 Elsevier B.V. All rights reserved. (d) Cell morphology on Mg scaffolds with (d1) 250 *μ*m and (d2) 400 *μ*m pore diameter following three days of incubation, as seen by SEM. (e) Viability of MG63 osteoblasts in (e1, e2) the control sample (culture medium without extracts), and the extractions of scaffolds with (e3, e4) 250 *μ*m and (e5, e6) 400 *μ*m pore diameter using live/dead assay after (e1, e3, e5) 1 and (e2, e4, e6) 3 d of incubation. (f) H&E staining of (f1, f2) subcutaneous and (f3, f4) muscular tissue in contact with scaffolds with (f1, f3) 250 and (f2, f4) 400 *μ*m pore diameter (the fibrous tissues that develop around the scaffolds are represented by the pentagrams). Reproduced from [205]. CC BY 4.0.

In the case of utilizing TWSH for fabricating Mg-based scaffolds, Jiang and He [204] prepared porous Mg (figure 9(a-a1)) [204] for orthopedic applications using a preform infiltration process. The researchers utilized Ti wires as the spacer, which were later removed through etching in an HF solution. Shrinkage cavity-like pores were located in the cylindrical sample’s center during microstructural evaluation (figure 9(a-a2)) that were attributed to the local high-density woven Ti wire arrangements. In addition, the microstructural analysis indicated that the porous morphology consisted of a pipe-like and linked pore structure, having a pore dimension of approximately 270 *μ*m, comparable to the size of the Ti wire (figure 9(a, a3–a5)). Moreover, the mechanical analysis of samples showed that with porosity ranging from 54% to 43%, the CYS of the samples was within the range of 4.3–6.2 MPa, while Young’s modulus ranges from 0.5 to 1.0 GPa [204]. In a related investigation, Li *et al* [149] used the same fabrication technique to manufacture porous Mg structures with multiple pore sizes and porosities. They characterized these structures in terms of damping capacities, compressive properties, and EA capabilities. Figure 9(b) shows a micrograph of a group of fabricated porous Mg samples in this study with 50% porosity and a pore diameter of 0.27 mm. In this investigation, two groups of Mg foams were fabricated. One group (figure 9(c, c1–c3)) had a constant porosity of 30% but different pore sizes (0.10, 0.27, and 0.40 mm), while the other group (figure 9(c, c4–c6)) had a constant pore diameter of 0.27 mm but varying porosities (30%, 40%, and 50%). Results indicated that the manufactured Mg foams showed substantial damping capacity and EA capabilities. It was shown that at a consistent 30% porosity, the loss factor and EAC increased as the pore diameter decreased. Moreover, at a consistent pore diameter of 0.27 mm, the loss factor rose while the EAC declined with increasing porosity. Additionally, the study revealed that porosity and pore diameter significantly impacted the relationship between damping capacity and temperature [149].

Further, Cheng *et al* [205] used the preform infiltration technique to manufacture two Mg scaffolds coated with MgF_2_. The scaffolds had interconnected pipe-like porosities and were fabricated using TWSH with 250 and 400 *μ*m diameters. The resulting Mg scaffolds had similar pore diameters and porosities of 55 ± 3% and 54 ± 3%, respectively. The evaluation of human MG63 osteoblast cell behavior towards the samples in this investigation revealed that the cells exhibit good growth and proliferation in the presence of the open-pore Mg scaffolds, which resulted in scaffolds having excellent cytocompatibility (figures 9(d) and (e)). Moreover, the effectiveness of both scaffolds in promoting the ingrowth of bone was confirmed through the increased activity of ALP and the expression of genes associated with osteogenic differentiation. Furthermore, *in vivo* animal studies demonstrated a mild local inflammatory response (figure 9(f)) and no evidence of systemic damage. In a rabbit model, it was observed that Mg scaffolds with larger pore sizes, while having the same porosity, can enhance vascularization and increase the expression of collagen type 1 and OPN, resulting in a rabbit model with increased bone mass and more mature bone development [205].

**Figure 10.**
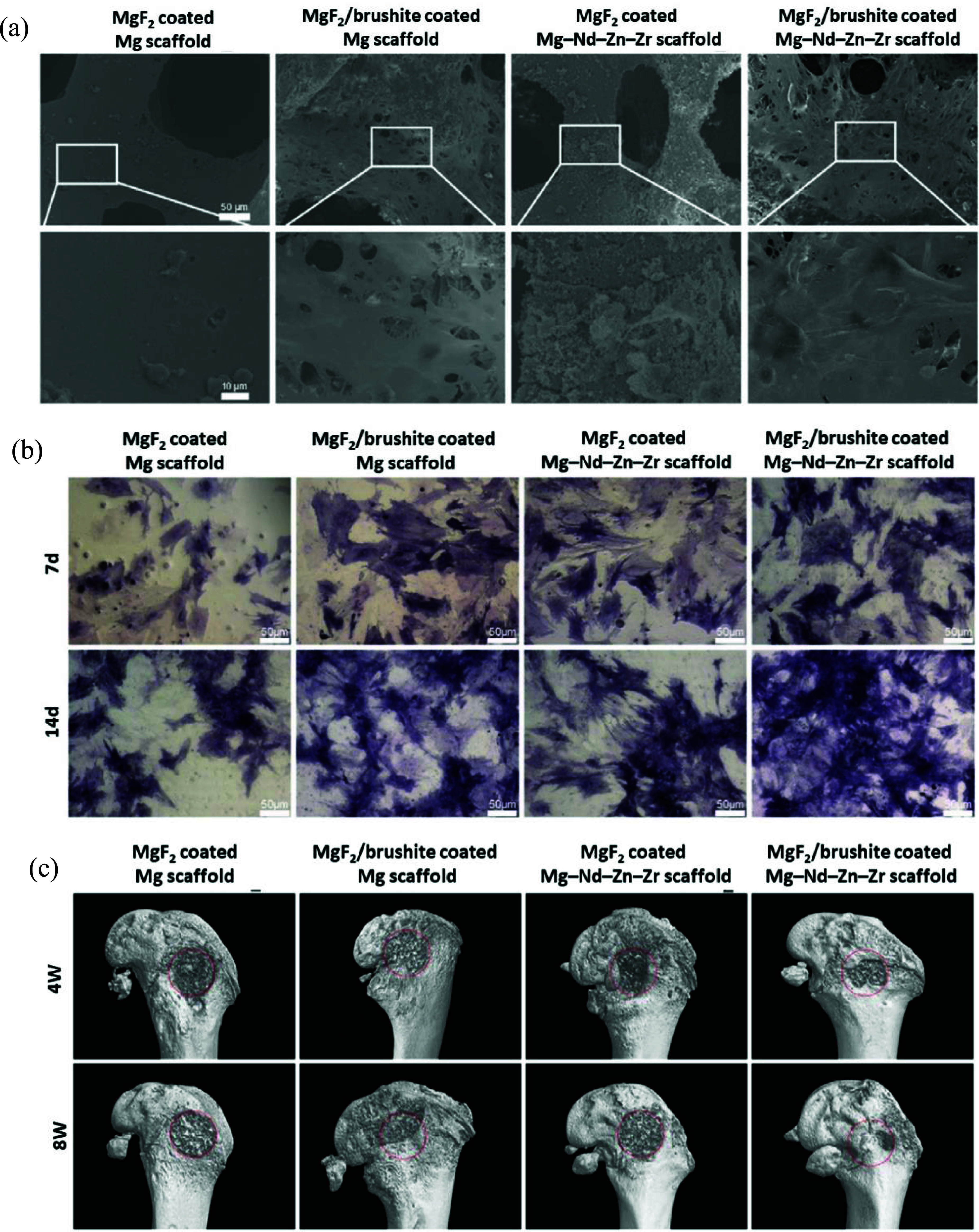
(a) *In vitro* cell adhesion of rBMSCs on scaffolds (The image within the white frame of the above panel is magnified in the panel below), (b) ALP staining of rBMSCs cultivated for 7 and 14 d using scaffold extract, and (c) representative 3D reconstructed micro-CT images showing the impact of various scaffolds on the new bone tissue formation within the defect location after 4 and 8 weeks (the red line encircled the location of the bone defect). Reprinted from [215], © 2020 Published by Elsevier Ltd.

Ti particles can also be used as a spacer material. For example, Wang *et al* [215] sintered 400–450 *μ*m spherical pure Ti particles into porous templates via a SPS system and subsequently infiltrated the templates with molten pure Mg and Mg–Nd–Zn–Zr alloy. Mg and Mg–Nd–Zn–Zr scaffolds coated with MgF_2_ were achieved after etching Ti templates with HF. Specimens were subsequently coated with brushite using the chemical deposition method. The *in vitro* assessment of the rBMSCs behavior toward samples indicated that the cell adhesion (figure 10(a)), proliferation rate, and ALP expression and activity (figure 10(b)) of MgF_2_/brushite-coated scaffolds (both Mg and Mg–Nd–Zn–Zr) surpassed those of MgF_2_-coated ones. Mg–Nd–Zn–Zr scaffold coated by MgF_2_/brushite demonstrated optimal cell in-growth and osteogenic differentiation, which notably enhanced mineralization, osteogenesis, and angiogenesis-related genes expression when cultured with rBMSCs [215]. The *in vivo* implantation of Mg–Nd–Zn–Zr scaffold coated by MgF_2_/brushite in rat and rabbit models demonstrated the effectiveness of the scaffold in stimulating angiogenesis, osteogenesis, and remodeling with the degradation of the scaffold, and precisely repairing the substantial bone defect (figure 10(c)).

### 2.4. Additive manufacturing

**Figure 11.**
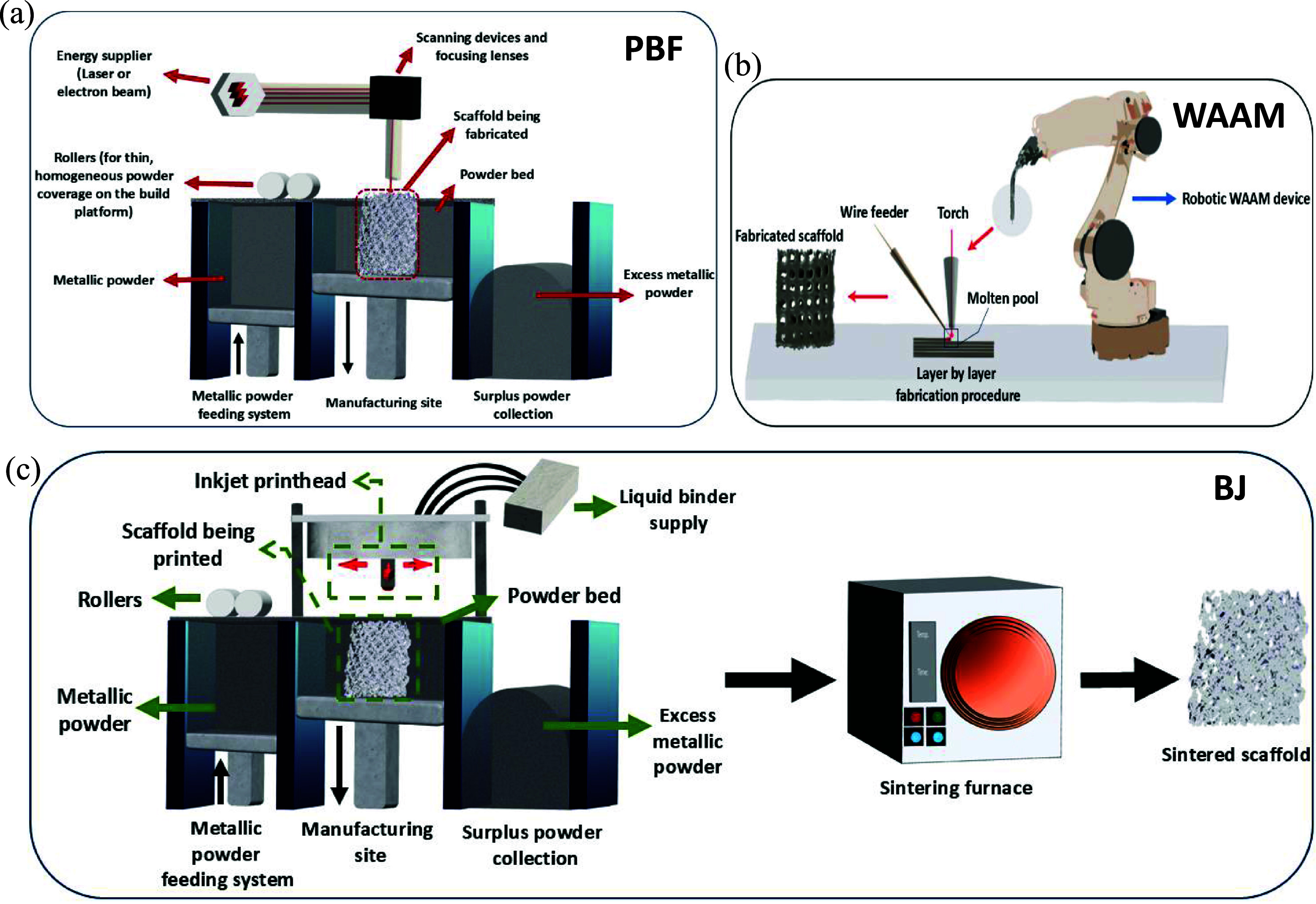
Schematic illustration of various AM techniques, including (a) powder bed fusion (PBF), (b) wire arc additive manufacturing (WAAM), and (c) binder jetting (BJ) for Mg-based scaffold fabrication.

AM is an advanced process that is transforming the manufacturing industry. It involves building 3D objects by adding material layer by layer, regardless of their shape or size. This method can be used to prototype and manufacture complex objects using metals, concrete, plastics, and even human tissue [216]. AM can produce intricate structures with features like interior pores and external shapes [217]. When making metallic structures using AM, a heat source such as an arc, electronic beam, or laser is used to selectively melt metal powder or wire along a specific path to create the desired components [218]. This study offers a brief overview of common AM techniques such as PBF, WAAM, and BJ in the fabrication of metallic scaffolds (figure 11). It also discusses their potential in the manufacturing of Mg-based scaffolds, which are comprehensively reviewed in the following sections.

#### 2.4.1. PBF.

**Figure 12.**
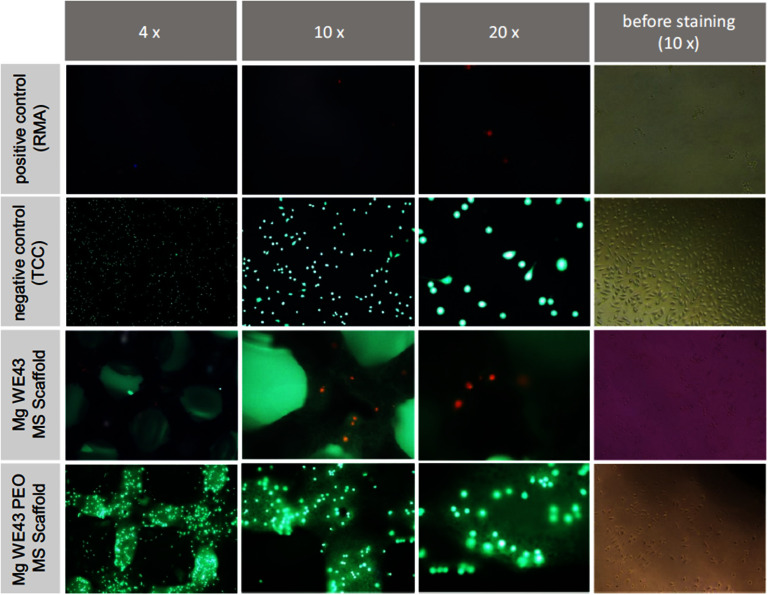
Using live dead staining (the stains that emit green and red light, respectively, to indicate living and dead cells), the viability of L929 mouse fibroblasts on WE43 Mg alloy scaffolds was assessed both with and without surface conversion using PEO. As reliable positive and negative controls, RMA and TCC were utilized. Reprinted from [232], © 2020 Elsevier B.V. All rights reserved.

PBF is an AM technique that involves fusing powdered material together using an energy source, such as a laser beam (referred to as L-PBF) or an electron beam (known as E-PBF). The process entails selectively melting or sintering powders to build solid structures layer by layer based on a computer-generated design. E-PBF, also called EBM, is not suitable for producing scaffolds made of Mg because the partial evaporation of Mg disrupts the electron beam inside the manufacturing chamber [219, 220]. In L-PBF, powder fusion can occur at temperatures above the melting points of the powders, termed as SLM, or at temperatures below the melting point through sintering, known as selective laser sintering (SLS) [219, 221–224]. SLM entails quick solidification cycles, resulting in refined grains and diminished compositional segregation, hence enhancing degradation resistance of Mg-based alloys [225, 226]. Moreover, studies have shown that the CYS of Mg-based alloys produced via SLM exceeds that of specimens manufactured through extrusion and casting of Mg-based alloys [227]. The SLM method is preferred for manufacturing Mg-based scaffolds over SLS, primarily because SLS results in lower densification and inferior mechanical characteristics in the produced Mg-based scaffolds [228]. Nonetheless, imperfections, like micro-holes and fractures in fabricating scaffold struts, along with residual stress, can readily occur throughout the SLM process [229]. Additionally, adjusting processing parameters, particularly energy density, is essential in the fabrication of Mg-based scaffolds to ensure complete melting of the powder without vaporization, given the narrow margin between the melting point of pure Mg (650 °C) and its boiling point (1090 °C) [230]. WE43 alloy is the most popular Mg-based alloy used in manufacturing Mg-based scaffolds and was extensively researched using the L-PBF (SLM approach) technique [231–235]. For example, Li *et al* [232] fabricated open-porous WE43 scaffolds with a body-centered cubic cell structure and varying strut diameters ranging from 250 to 750 *μ*m using the L-PBF technology. After the fabrication process, the researchers further adjusted the microstructure of the scaffolds through thermal solution and aging heat treatments. A PEO coating was applied to modify the surface of multiple specimens. Biocompatibility assessments of the samples on L929 mouse fibroblasts (figure 12) revealed that scaffolds lacking PEO surface treatment exhibited some cytotoxicity when exposed to cells or culture media. Additionally, after PEO treatment of the scaffolds, samples showed improved compatibility with cells by forming a surface that promotes adhesion and minimizes the release of degradation by-products [232].

**Figure 13.**
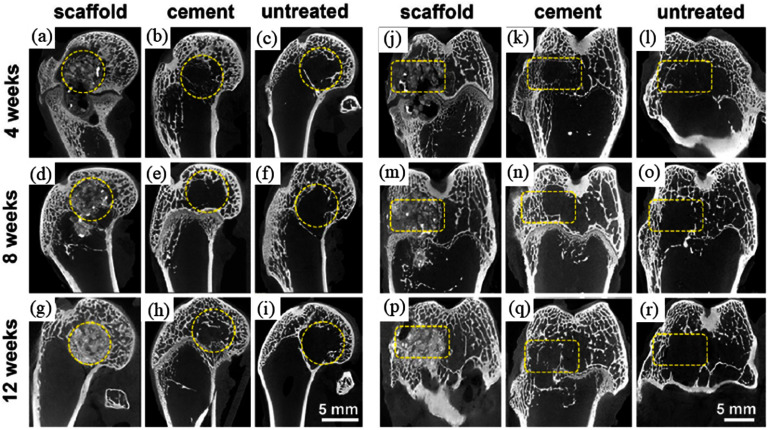
The subsequent to surgery sagittal and coronal Micro-CT scans of various groups were taken at various times intervals after the operation. The yellow circles and boxes highlight the areas of the predetermined femoral condyle defect. Reproduced from [233]. CC BY 4.0.

Next, Liu *et al* [233] studied how the processing parameters of L-PBF affect the quality of WE43 porous scaffolds. They investigated the properties of the WE43 porous scaffolds through *in vitro* and *in vivo* studies. The results showed that by controlling the porous design, it is possible to produce WE43 Mg-based scaffolds with a wide range of CSs (4.4–23.5 MPa) and Young’s moduli (154–873 MPa). Additionally, improved osteogenic effects of WE43 scaffolds were observed after 8- and 12-week implantation in rabbits compared to those filled with calcium sulfate bone cement and untreated groups (figure 13) [233]. In another study, Yao *et al* [236] utilized SLM to alter the surface properties of two distinct Mg-based alloys (Mg–0.6Ca and Mg–0.5Zn–0.3Ca) to improve their corrosion resistance and enhance their surface microhardness. The results not only showed that the samples’ microhardness and corrosion resistance were improved but also indicated that the adhesion and spread of MG63 cells on the surface of specimens were adequate due to good biocompatibility.

#### 2.4.2. WAAM.

The WAAM process involves continuously feeding a metal wire at a consistent pace, which is then melted by an electric arc and applied onto a substrate or the existing deposition layers. WAAM has a significantly greater deposition rate, increased material utilization, and is less expensive compared to PBF-based techniques. Working within a chamber is unnecessary because the welding gun provides local shielding. Therefore, there are virtually no restrictions on the dimensions of the part [218, 237, 238]. Robotic WAAM can be considered as the most novel and precise form of WAAM. WAAM for Mg-based alloys enables the production of sizable scaffolds with optimal material usage, eliminating the requirement to manipulate powders or vast volumes of molten Mg. Additionally, WAAM can manufacture Mg-alloys containing intermetallic microstructural constituents [218, 239, 240]. However, WAAM also has notable limitations, such as the development of residual stress in the printed structure due to the application of high temperature and subsequent rapid solidification, as well as inadequate surface finish quality in net-shape printed components such as scaffolds. One major limitation of WAAM for Mg-based alloys printing is the lack of sufficient filler wire availability [239, 241–243]. WAAM has been mainly utilized for the fabrication of Mg-based alloys such as AZ31 [218, 244], AZ91D [243], and AZ61A [242] in non-porous forms.

#### 2.4.3. Binder jetting.

**Figure 14.**
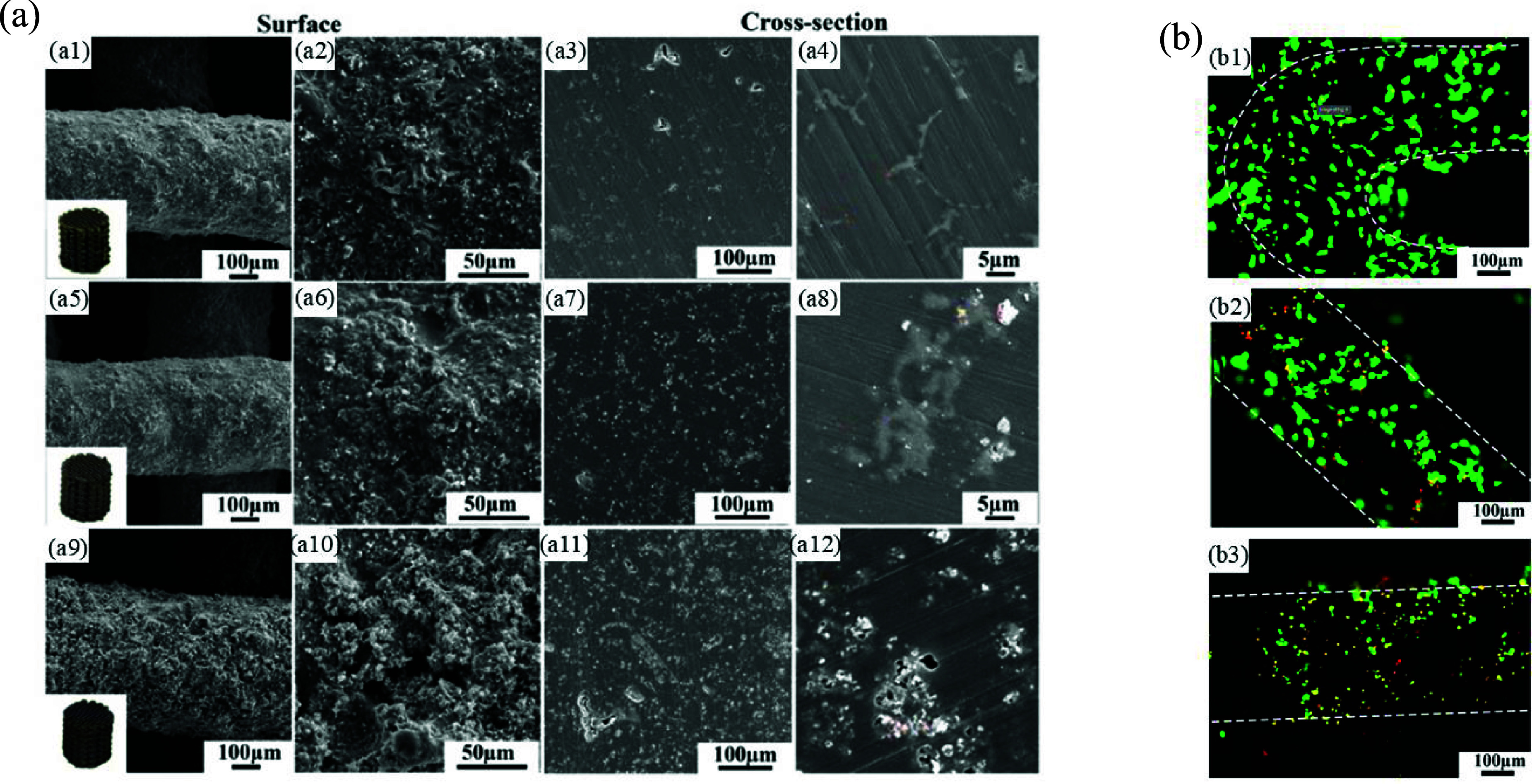
(a) Microstructures: (a1–a4) Mg–Zn/5TCP, (a5–a8) Mg–Zn/10TCP, (a9–a12) Mg–Zn/15TCP. (b) The *in vitro* assessment of the Mg–Zn/5TCP and Mg–Zn/10TCP samples using ethidium homodimer-1 (red, indicating harmed cells) and calcein acetoxymethyl (green, indicating viable cells) fluorescence staining of MC3T3-E1 preosteoblasts after three days of culture on Mg–Zn/10TCP (b1), Mg–Zn/5TCP (b2), and Mg–Zn (b3), respectively (the rough margins of the scaffold struts are indicated by the white dashed lines). Reproduced with permission from [206]. CC BY-NC-ND 4.0.

BJ, also known as BJP, is a versatile powder-based 3D printing method that can use a variety of materials, including ceramics, polymers, metals, and composites. Unlike other AM technologies, BJ does not require the creation of support structures [245–248]. The BJ process involves two steps. In the first step, the powder is bound together with a binder to produce a green component based on the CAD design. In the subsequent step, the excess powders are removed, and then the green component undergoes sintering processes to remove the binder material and produce the final fabricated components [248]. There is a possibility of fabricated part shrinkage during the sintering stage [249]. BJ is known for its cost-effective machinery, its ability to use various materials, and the option to stack components during production [206, 250, 251]. This method allows for the manufacturing of highly interconnected porous structures made of Mg with various pore sizes. The technique involves layer-by-layer construction and can adjust the pore morphology after printing by changing the sintering specifications [251–253]. Surface finishes of scaffolds manufactured by BJ can offer increased roughness in comparison to scaffolds manufactured by other AM methods, which might demand additional post-processing procedures, like machining or polishing, to attain the appropriate surface quality [249]. For example, Dong *et al* [206] fabricated composite scaffolds using porous Mg–Zn/*x β*-TCP materials (figure 14(a)), where *x* represents weight percentages of 5, 10, and 15. These scaffolds were designed to treat bone defects using the BJ approach. The assessment of scaffold degradation revealed that the Mg–Zn/5TCP samples exhibited a slower pace of *in vitro* biodegradation (0.5 mm yr^−1^) compared to Mg–Zn sample. The biodegradation behavior of the Mg–Zn/15TCP specimens was negatively affected by the uneven dispersal of *β*-TCP particles. Furthermore, the cytocompatibility toward MC3T3-E1 preosteoblasts (figure 14(b)) [206] and ALP activity of Mg–Zn/5TCP and Mg–Zn/10TCP specimens were significantly enhanced compared to Mg–Zn specimens [206]. Table 4 summarizes the advantages and disadvantages of the prevalent non-AM and direct AM approaches pertinent to the fabrication of Mg-based scaffolds, as presented in this study.

**Table 4.** Summary of the advantages and disadvantages of non-AM and direct AM approaches in Mg-based scaffold fabrication according to the description in this study.

Fabrication technique	Advantages	Disadvantages	References
GASAR	Enables accurate regulation of the porosity, pore size, and distribution of the oriented pore structure.Particularly useful for creating scaffolds with high porosity levels.	Possibility of the formation of unwanted substances inside the scaffold structure due to the reaction between the molten Mg-based alloy and the available gas.Challenging precise control over processing conditions.	[111, 116–118]

Investment casting	High structural accuracy and exceptional surface finishes.Particularly useful for creating scaffolds with interconnected porosities.	Possibility of the molten Mg-based alloy not fully penetrating all parts of the mold due to narrow pathways and the presence of air.The reactions between the Mg-based melt and the mold can produce a non-desired oxide layer on the part surface.	[106, 131–135, 138, 139]

Space holder	Being able to fabricate a broad range of Mg-based scaffolds in terms of pore geometry (either consistent porous structures or with varied pore size and/or porosity) and interconnectivity.No high-tech equipment is required.	Leaching, one of the most frequent ways to remove space holders, can corrode Mg-based scaffolds.The potential for the Mg-based alloy to interact with space holders during the fabrication procedure. Possibility of remaining space holders in the scaffold structure due to incomplete removal, causing undesired features.	[4, 169, 174, 190–194]
L-PBF (SLM)	Fast solidification refines grains and improves Mg-based scaffold degradation resistance and mechanical performance. Being able to fabricate Mg-based scaffolds with high structural accuracy and delicacy.	SLM process can easily cause micro holes, cracks, and residual stress.Challenging adjustment of processing parameters such as energy density of the laser beam.	[225–230]

WAAM	WAAM outperforms PBF-based techniques in deposition rate, material utilization, and cost.Local shielding from the welding gun inhibits oxidation.	High temperature and quick solidification cause residual stress in printed structures.Inadequate surface finish quality in net-shape printed components such as scaffolds.	[218, 237–243]

Binder jetting	Cost-effective machinery	Surface finishes of scaffolds manufactured by BJ can offer increased roughness in comparison to scaffolds manufactured by other AM methods.Capable of fabricating Mg-based scaffolds with a good degree of delicacy and precision. Possibility of fabricated part shrinkage during the sintering stage.	[206, 249–253]

#### 2.4.4. Indirect AM.

**Figure 15.**
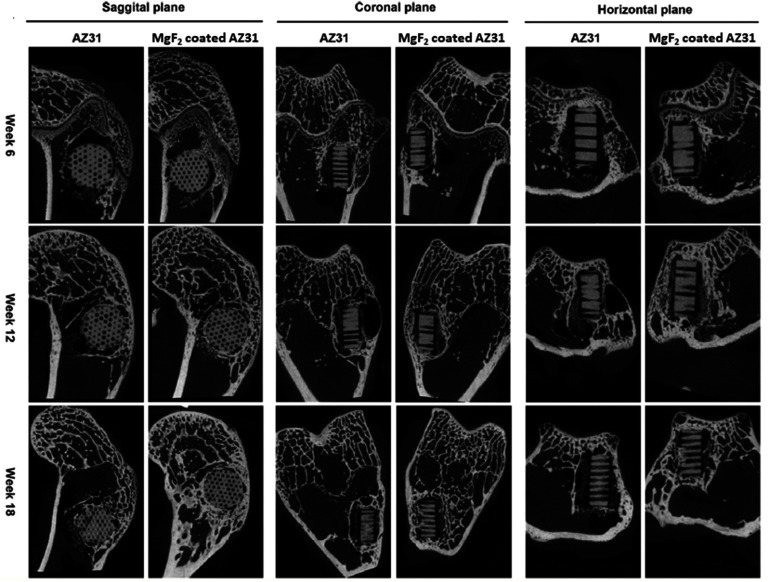
Micro-CT reconstructed pictures of the femoral condyles implanted with the AZ31 or MgF_2_ coated AZ31 scaffolds, demonstrating bone regrowth and scaffold degradation at weeks 6, 12, and 18 after implantation. Reprinted from [214], © 2016 Elsevier B.V. All rights reserved.

Indirect AM techniques involve using AM methods such as FDM to craft polymeric scaffold patterns for casting and infiltration methods or employing subtractive manufacturing methods such as laser perforation. Laser perforation is the process of directing a concentrated laser beam onto the surface of a material. This technique utilizes the energy absorbed to locally melt or vaporize the material, generating pores. By utilizing a multifunctional laser processing machine on casted Mg-based ingots, this technique can produce Mg-based stents and bone scaffolds with interconnected porosity structures, providing control over pore morphology, size, porosity, and mechanical strength [214, 254]. For example, Geng *et al* [254] developed porous Mg scaffolds for use as cancellous bone scaffolds in weight-bearing situations. Compression tests showed that the porous Mg scaffolds exhibited significantly improved mechanical properties, attributed to the lower quantity of defects and voids in the struts resulting from the superior laser perforation technique. In another study, Yu *et al* [214] effectively manufactured AZ31 scaffolds coated with MgF_2_ using laser perforation and fluoride treatment. The AZ31 scaffolds coated with MgF_2_ demonstrated superior corrosion resistance, osteogenic activity, and better cytocompatibility compared to the uncoated AZ31 scaffolds. Furthermore, *in vivo* investigation on the healing of the femoral condyle defects (figure 15) demonstrated that the MgF_2_-coated AZ31 scaffolds exhibited better osteoconductive and osteoinductive capabilities than the uncoated AZ31 scaffolds in restoring femoral condyle defects [214].

As mentioned before, the fast CR of Mg and Mg-based alloys in physiological settings presents obstacles to their effective utilization as implants, particularly in orthopedic applications due to a rapid loss in mechanical stability. To address this, it is recommended to use elements as alloying additions or coatings to enhance both the corrosion resistance [255–258]. Table 5 provides the corrosion performance of Mg-based alloy scaffolds manufactured using various described fabrication techniques, along with corresponding notes regarding their corrosion properties.

**Table 5.** A comprehensive summary of the corrosion characteristics and remarks related to the Mg-based scaffolds that have been produced using different manufacturing methods.

Fabrication technique	Material/Corrosion environment (CE)	Porosity (%)/pore diameter	*E*_corr_ (V)	*I*_corr_ (A cm^−2^)/Corrosion rate (mm year^−1^)	Remarks	References
Space holder (P/M technique)	Pure Mg (CE = SBF)	—	–1845 ± 13 mV_SCE_	41 ± 3 *μ*A·cm^−2^	A small amount of Ca present within the composite scaffold aids to sustain brucite (Mg(OH)_2_). This can serve as a barrier and reduce the rate at which SBF penetrates the substrate, preventing the direct contact with the Mg alloy.Loading doxycycline antibiotic from 1%–10% in the Mg-1 wt.% Ca-10 Wt.% TiO_2_ scaffold does not change degradation rate significantly.	[208]
	Mg- 1Ca—10TiO_2_ alloy (CE = SBF)	65%–67% PS = 600–800 *μ*m	–1733 ± 11 mV_SCE_	211 ± 10 *μ*A·cm^−2^		

Space holder (P/M technique)	Pure Mg (CE = SBF)	63%–65% PS = 600–800 *μ*m	–1811 mV_SCE_	213 *μ*A∙cm^−2^	Zinc (Zn) in the Mg–Zn scaffold has a number of consequences.The production of brucite (Mg(OH)_2_) is facilitated by zinc and serves as a barrier by postponing the diffusion of SBF into deeper scaffold layers and subsequently Zn replaces Mg^2+^ cations in Mg(OH)_2_ in SBF solution, creating a shielding layer on the alloy surface. Zinc Oxide Layer: Zinc passivates the scaffold surface by forming a zinc oxide layer on the Mg–Zn alloy’s surface. The corrosion behavior of MZ-TC scaffolds in SBF is not appreciably affected by tetracycline concentrations ranging from 1 to 10%.	[201]
	Mg-6 wt.% Zn (CE = SBF)	63%–65% PS = 600–800 *μ*m	–1741 mV_SCE_	47 *μ*A∙cm^2^		
Space holder (P/M technique)	Mg-6 wt.% Zn with 0% NH_4_HCO_3_ as space holder (CE = Hanks)	6.7 vol.% PS = 32.3 *μ*m	—	CR = 6.5 mg cm^−2^ d^−1^	A rise in space-holder particle amount, particularly when it exceeded 20%, had a detrimental effect on the Mg-6 wt.% Zn scaffolds’ ability to resist corrosion.The solution’s pH elevated quickly for all samples when the Mg-6 wt.% Zn scaffolds soaked in Hanks’ solution for up to 168 h, peaking as early as 24 h after immersion. The porous magnesium-6 wt.% zinc scaffolds degraded too quickly and had a shorter lifespan than 12 weeks, which is not long enough for full bone repair.	[259]
	Mg-6 wt.% Zn with 10% NH_4_HCO_3_ as space holder (CE = Hanks)	17.3 vol.% PS = 133.6 *μ*m	—	CR = 10.3 mg cm^−2^ d^−1^		
	Mg-6 wt.% Zn with 20% NH_4_HCO_3_ as space holder (CE = Hanks)	28.6 vol.% PS = 202.5 *μ*m	—	CR = 16.1 mg cm^−2^ d^−1^		
	Mg-6 wt.% Zn with 30% NH_4_HCO_3_ as space holder	40.1 vol.% PS = 286.8 *μ*m	—	CR = 26.7 mg cm^−2^ d^−1^		
	Mg-6 wt.% Zn with 40% NH_4_HCO_3_ as space holder (CE = Hanks)	52.2 vol.% PS = 384.2 *μ*m	—	CR = 37.4 mg cm^−2^ d^−1^		

Space holder (Preform infiltration)	Mg–Nd–Zn–Zr scaffold coated by HF (CE = DMEM)	∼75% The average main PS = ∼450 *μ*m The average interconnected PS = ∼200 *μ*m	—	CR = ∼0.29	Scaffolds that were coated had an initial degradation ratio that was approximately 81% lower than those that were not.	[260]
	Mg–Nd–Zn–Zr scaffold coated by HF and subsequently by brushite/Ag_3_PO_4_ (CE = DMEM)	∼68% The average main PS = ∼400 *μ*m The average interconnected PS = ∼140 *μ*m	—	CR = 0.10–0.15	In the beginning stages of immersion, the inclusion of Ag_3_PO_4_ particles has no unfavorable effects on the degradation behavior of Mg-based scaffolds coated with HF and brushite; but, after 14 d of immersion, their corrosion resistance may somewhat decline.	
TWSH (Preform infiltration)	Pure Mg coated with MgF_2_ (CE = DMEM)	—	–1.56	5.44 × 10^−5^ CR = 2.4 ± 0.2	Zn and Nd alloying enhanced the porous Mg specimen’s resistance to corrosion.Mg alloy can improve corrosion resistance by strengthening solid solution into the matrix and lowering potential difference when alloyed with Nd.	[261]
	WE43 coated with MgF_2_ (CE = DMEM)	—	–1.50	1.25 × 10^−5^ CR = 1.7 ± 0.2		
	Mg-6.78 wt%Nd-0.33 wt%Zn coated with MgF_2_ (CE = DMEM)	—	–1.41	6.28 × 10^−6^ CR = 0.83 ± 0.06		

TWSH (Preform infiltration)	Pure Mg scaffolds coated with MgF_2_ (CE = DMEM containing 10% FBS)	55 ± 3 PS = 250 *μ*m	—	CR = 1.3 ± 0.1	Based on differences in pH levels and reductions in mass of specimens, during the first seven days, the number of Mg ions appears to be similar in Mg scaffolds with varying pore sizes. But with extended immersion periods the Mg scaffold with lager pore size (400 *μ*m) seems to degrade faster.The existence of an MgF_2_ coating layer enhances the degradation behavior of Mg scaffolds.	[205]
	Pure Mg scaffolds coated with MgF_2_ (CE = DMEM containing 10% FBS)	54 ± 3 PS = 400 *μ*m	—	CR = 1.5 ± 0.2		

AM/SLM	ZK30 (CE = SBF)	—	–1.57	0.10 (mA cm^−2^) CR = 3.7 ± 0.1	The SLMed ZK30–0.6GO exhibited the least corrosion pace due to the combined effects of grain refinement and reduced presence of MgZn_2_ particles resulting from increased GO content, which counteracted the potential increase in corrosion rate associated with higher GO content.	[262]
	ZK30–0.3GO (CE = SBF)	—	–1.59	0.33 (mA cm^−2^) CR = 10.8 ± 0.1		
	ZK30–0.6GO (CE = SBF)	—	–1.65	0.10 (mA cm^−2^) CR = 3.4 ± 0.1		
	ZK30–0.9GO (CE = SBF)	—	–1.51	0.49 (mA cm^−2^) CR = 15.6 ± 0.1		

CR = Corrosion rate; CE = Corrosion environment.

## 3. Conclusions

Mg-based scaffolds are considered very promising as biodegradable metallic materials for tissue engineering, bone defect and fracture scaffolding, and fixation. This detailed overview summarizes the key manufacturing methods used to manufacture porous Mg-based structures, including the GASAR technique, pattern casting (specifically IC), techniques using solid space holders, and AM. The paper also provides an overview of the most significant findings regarding the microstructure specifications, mechanical properties, corrosion behavior, antibacterial activity, cell behavior, cytotoxicity evaluations, and *in vivo* investigations of Mg-based scaffolds. The following are specific conclusions on key manufacturing techniques that we drew from the literature review:

(1) The **GASAR technique** for manufacturing Mg-based scaffolds offers several benefits. These include constructing scaffolds with an oriented porosity structure with smooth pore walls. This mimics the natural internal structure of bones and improves the mechanical stability and loading properties along the length of the pores. As a result, these scaffolds are ideal for applying in bone tissue engineering; however, since most of the research based on utilizing the GASAR technique is focused on Mg–Ag alloys [126–128], more investigations for achieving optimum processing parameters for other Mg-based alloys are required.
(2) **IC** is highly effective in producing intricate and precise porous scaffold structures of Mg-based alloys. These structures are ideal for scaffolds in tissue engineering applications.
(3) Fabrication techniques based on solid space holders can be divided into two main fabrication techniques: P/M and melt infiltration. NaCl is one of the most common spacers broadly used to fabricate scaffolds made of Mg-based alloys. Scaffolds with cuboidal and spherical pore morphologies can be produced using NaCl spacers. The main drawbacks of utilizing NaCl particles as spacers are the possibility of fabricated scaffold corrosion during the spacers leaching (which can be controlled by using inhibitor agents such as potassium permanganate [164]) and the possibility of increasing the CR of the fabricated Mg-based scaffolds due to the remained spacers in the structure due to the incomplete leaching procedure. Carbamide, camphane, and PMMA are other space holders that can be utilized for manufacturing Mg-based scaffolds, which eliminate the need for an aqueous solution for leaching spacers and can be extracted from the structure by thermal decomposition or sublimation (specifically for camphane), reducing the risk of Mg-based scaffold corrosion during the spacers’ leaching process. Ti wires can be considered a more novel space holder that provides the possibility of fabricating Mg-based scaffolds with cellular porosity structure, which mimics the natural structure of the cancellous bone and provides the possibility of achieving and regulating the mechanical properties of the Mg-based scaffolds by changing the orientation of porosities without sacrificing the required porosity [205]. In addition, a MgF_2_ coating layer is formed on the surface of Mg-based scaffolds during the TWSH etching in HF solution, which not only can increase the Mg-based scaffolds’ resistance to corrosion in biological environments but also improve the cytocompatibility, osteogenic activity, osteoconductivity, and improved osteoinductive properties of Mg-based scaffolds.
(4) The utilization of AM appears to be the most suitable approach for producing porous structures of Mg-based alloys. The best suitable technology is the L-PBF techniques, which encompass SLM and SLS and do not require sacrificial materials, such as binders, as shown in the BJ and indirect AM approaches. EBM is not a suitable technique for manufacturing scaffolds made of Mg due to the evaporation of Mg during manufacturing. Scaffolds created using SLM technology exhibit exceptional qualities, including the absence of porosity in struts and a high level of densification. The use of SLM results in the production of components with refined grains, increased alloying elements retained in solid solution and a uniform distribution of microstructural constituents. These characteristics are attributed to an increased rate of cooling during alloy solidification.
(5) The fundamental objective of all modification techniques is to decrease the rate at which Mg substrates corrode, including **alloying** (with ions such as Zn and Zeo) and applying **coating** techniques. It was demonstrated that utilizing various polymeric- and ceramic-based coatings can greatly enhance the degradation resistance of Mg-based scaffolds. Furthermore, since poor adhesion can encounter challenges from early detachment or the infusion of corrosive fluids, adhesion strength is also a critical factor impacting the protective duration and efficiency of applied coatings. Furthermore, it was revealed that applying PEO coating can enhance the corrosion resistance of porous Mg-based structures and be regarded as a surface modification to improve the adhesion strength of polymeric coatings. Applying multifunctional coatings such as TA or ceramic-based coatings on the surface of Mg-based scaffolds can increase the scaffolds’ resistance to corrosion and improve their biocompatibility and cell behavior.

## 4. Future perspectives

The exploration of porous Mg-based structures for medical applications is still in its early stages, requiring further work to design and manufacture medical implants. Here are a few suggestions:

(1) The formation of intermetallic phases during the fabrication of Mg-based porous scaffolds is poorly investigated in spite of the fact that intermetallic phases accelerate metal corrosion in a biological environment and affect its mechanical strength. Hence, more investigation is necessary to ascertain these intermetallic phases’ precise chemical compositions, quantities, and impacts on the ultimate characteristics of the produced Mg-based porous structures.
(2) The changes in the mechanical strength of Mg-based scaffolds throughout the degradation test is another key factor that has not been reported in the literature sufficiently. This topic, which is key to the implementation of Mg-based scaffolds, warrants further investigation [106, 107, 142, 153, 196, 203].
(3) Additional research is necessary to determine the optimal processing parameters for each Mg-based alloy in terms of fabrication procedures. Furthermore, exploring innovative techniques and new composite materials for Mg-based scaffold surface treatment is crucial to enhance further the degradation behavior and physiological capabilities of scaffolds and implants made of Mg.
(4) It is crucial to consider potential differences in the biological responses of Mg-based scaffolds under laboratory testing and *in vivo* circumstances. While *in vitro* studies have advanced significantly, there is a scarcity of *in vivo* and clinical investigations on the biodegradation performance and biocompatibility behavior of manufactured Mg-based scaffolds. Therefore, further investigation is necessary to evaluate the *in vivo* and clinical efficacy of scaffolds made from Mg.
(5) More research is needed on creating Mg-based scaffolds that are infused with various pharmaceuticals, such as antibiotics and growth factors (like bone morphogenetic proteins, insulin-like growth factors, and vascular endothelial growth factors). The research should focus on determining the best dosages for loading these pharmaceuticals onto the scaffolds in order to improve their antibacterial properties, ability to support bone growth, and ability to stimulate bone formation.
(6) Additionally, it is essential to further investigate the development of biocompatible radiopaque markers to enhance the radiopacity of low-density Mg-based scaffolds, such as stents.
